# Identification of Root-Associated Bacteria That Influence Plant Physiology, Increase Seed Germination, or Promote Growth of the Christmas Tree Species *Abies nordmanniana*

**DOI:** 10.3389/fmicb.2020.566613

**Published:** 2020-11-17

**Authors:** Adriana M. Garcia-Lemos, Dominik K. Großkinsky, Saqib Saleem Akhtar, Mette Haubjerg Nicolaisen, Thomas Roitsch, Ole Nybroe, Bjarke Veierskov

**Affiliations:** ^1^Department of Plant and Environmental Sciences, Faculty of Science, University of Copenhagen, Frederiksberg, Denmark; ^2^Bioresources Unit, Center for Health and Bioresources, AIT Austrian Institute of Technology GmbH, Tulln an der Donau, Austria; ^3^Department of Adaptive Biotechnologies, Global Change Research Institute, Brno, Czechia

**Keywords:** PGPR, *Bacillus*, *Paenibacillus*, phytohormones, plant carbohydrates, antioxidative enzymes, rhizosphere

## Abstract

*Abies nordmanniana* is used for Christmas tree production but poor seed germination and slow growth represent challenges for the growers. We addressed the plant growth promoting potential of root-associated bacteria isolated from *A. nordmanniana*. Laboratory screenings of a bacterial strain collection yielded several *Bacillus* and *Paenibacillus* strains that improved seed germination and produced indole-3-acetic acid. The impact of three of these strains on seed germination, plant growth and growth-related physiological parameters was then determined in greenhouse and field trials after seed inoculation, and their persistence was assessed by 16S rRNA gene-targeted bacterial community analysis. Two strains showed distinct and significant effects. *Bacillus* sp. s50 enhanced seed germination in the greenhouse but did not promote shoot or root growth. In accordance, this strain did not increase the level of soluble hexoses needed for plant growth but increased the level of storage carbohydrates. Moreover, strain s50 increased glutathione reductase and glutathione-S-transferase activities in the plant, which may indicate induction of systemic resistance during the early phase of plant development, as the strain showed poor persistence in the root samples (rhizosphere soil plus root tissue). *Paenibacillus* sp. s37 increased plant root growth, especially by inducing secondary root formation, under in greenhouse conditions, where it showed high persistence in the root samples. Under these conditions, it further it increased the level of soluble carbohydrates in shoots, and the levels of starch and non-structural carbohydrates in roots, stem and shoots. Moreover, it increased the chlorophyll level in the field trial. These findings indicate that this strain improves plant growth and vigor through effects on photosynthesis and plant carbohydrate reservoirs. The current results show that the two strains s37 and s50 could be considered for growth promotion programs of *A. nordmanniana* in greenhouse nurseries, and even under field conditions.

## Introduction

The genus *Abies* comprises about 40 species that predominantly occur in boreal and subalpine forest zones (Liu, 1971). Commercially, *Abies* spp. have an important value, particularly in the Northern Hemisphere, where different *Abies* species are grown for the production of wood, pulp and paper (Mercado-Blanco et al., 2018) and for use as Christmas trees (Nielsen et al., 2011). According to the Christmas Tree Growers Council of Europe (CTGCE), around 120,000 hectares are planted with Christmas trees in Europe^1^, and of the 75 million trees that are sold each year, 50 million belong to the species *Abies nordmanniana* (Stev.)^2^.

The natural slow growth of *A. nordmanniana* represents a challenge to the growers as the trees only reach their harvesting stage after 10–13 years (Nielsen et al., 2011). Furthermore, it has been shown that seeds of many *Abies* species require a long period of cold stratification to break dormancy and enable germination (Zulueta-Rodríguez et al., 2015), and that a correct root and seedling development is essential for survival and establishment of Christmas trees after the nursery stage (Seifert, 2015). To overcome the slow growth of *A. nordmanniana*, commercial chemical plant growth regulators have been applied (Rasmussen et al., 2009; Nielsen et al., 2011), but are being out-phased due to concerns about their impact on human health and the environment (Sørensen and Danielsen, 2006). To lower the production time of *A. nordmanniana*, new approaches for promotion of growth and seed germination are thus of utmost importance for the Christmas tree industry.

Symbiotic interactions between plants and microorganisms have been widely employed to improve plant growth. The plant rhizosphere harbors plant growth promoting rhizobacteria (PGPR) constituting a wide and diverse group, which is able to enhance plant growth and development by various mechanisms (Vessey, 2003; Martínez-Viveros et al., 2010; Glick, 2012; Olanrewaju et al., 2017). PGPR can produce phytohormones like auxins and cytokinins that can enhance plant growth (Wu et al., 2009; Großkinsky et al., 2016; Kang et al., 2019), and they can improve plant nutrition by fixing nitrogen (Bal and Chanway, 2012) and by solubilizing nutrients such as phosphorus (Lucy et al., 2004; Glick, 2012). Further, PGPR can suppress plant pathogens with the production of antimicrobial metabolites, by competition for nutrients such as iron, and by inducing plant systemic defense responses to pathogen attack (Ortíz-Castro et al., 2009; Govindasamy et al., 2010; Glick, 2012; Bakker et al., 2013; Grady et al., 2016; Olanrewaju et al., 2017; Kumari et al., 2019). Finally, PGPR can induce plant resistance to abiotic stressors such as drought and high salinity (Lata and Gond, 2019).

The effects of PGPR on plant growth are frequently determined by measuring plant biomass, but their effects may even be revealed by measuring constituents or processes underlying plant growth, e.g., chlorophyll levels. Chlorophyll levels are central for plant growth and have previously been reported to increase after inoculation with PGPR in other plants, including conifers (Xie et al., 2009; Aghai et al., 2019). In the case of *A. nordmanniana*, the chlorophyll level is also an important measure as greenness is a crucial quality trait for the growers.

Trees generally store large amounts of freely available carbon reserves, commonly termed non-structural carbohydrates (NSC) in the form of soluble sugars and the storage molecule starch (Weber et al., 2019). Sucrose is the form in which most carbon is transported in the plant (El-Lithy et al., 2010), whereas small size monosaccharides are actively used for growth (Schiestl-Aalto et al., 2019). Hence, the concentration and distribution of NSC is indicative of the accumulated carbohydrate reserves available for growth, and can be affected by bacterial inoculants, even sometimes without affecting growth (Vardharajula et al., 2011; Gagné-Bourque et al., 2016; Marcos et al., 2016), indicating that carbohydrate levels may be sensitive markers of inoculant impact on plant physiology.

PGPR can influence the activity of plant antioxidative enzymes (Helepciuc et al., 2014). Enzymes such as superoxide dismutase, catalase and peroxidase are essential for scavenging the reactive oxygen species (ROS) superoxide and hydrogen peroxide, respectively. Other enzymes like ascorbate peroxidase, monodehydroascorbate reductase, dehydroascorbate reductase, and glutathione reductase are part of the ascorbate-glutathione cycle that mediates reduction of the non-enzymatic ROS scavengers ascorbate and glutathione (Das et al., 2015; Caverzan et al., 2016). ROS are produced by metabolic processes and in particular accumulate during abiotic stress (Mhadhbi et al., 2004; Jebara et al., 2005; Sandhya et al., 2010a,b; Ghorbanpour et al., 2013; Bharti et al., 2016), or are produced by plants in response to biotic stress (Caverzan et al., 2016; Rais et al., 2017). The ability of a PGPR to increase the plants antioxidative defense capacity would consequently reflect a potential for alleviation of biotic or abiotic stress that plants may encounter in a variable soil environment.

The application of PGPR in silviculture and conifer crop production is not a common practice (São José et al., 2019). However, few studies have demonstrated positive effects of PGPR inoculation on growth or seed germination of forest tree species (Zulueta-Rodríguez et al., 2015; Rostamikia et al., 2016; São José et al., 2019), but it remains an open question if bacteria associated with roots of *A. nordmanniana* exhibit growth promoting properties.

For the current study, we hypothesized that the bacterial community associated with roots of *A. nordmanniana* contains bacteria that can promote plant growth and affect growth-related physiological parameters. In consequence, the aims of this study were: (1) to isolate root-associated bacteria which stimulate growth and seed germination of *A. nordmanniana* under greenhouse and field conditions; (2) to determine if growth stimulation correlated to bacterial influences on plant carbohydrate and chlorophyll levels; and (3) to determine if selected isolates affected plant signatures of antioxidative enzyme activities indicative for their ability to alleviate biotic or abiotic stress.

## Materials and Methods

### Sampling Site and Collection of Plant Material

One-, two- and three-year-old plants of *A. nordmanniana* were collected during July 2016 from three Christmas tree nurseries; sampling site 1: the field nursery Primo Plant Ejendomme ApS, located in Hadsund, Denmark (56°44′22.7″N 10°03′36.7″E); sampling site 2: the field nursery Baumschule Engler, located in Hohenlockstedt, Germany (53°58′30.5″N 9°37′51.9″E); and sampling site 3: the greenhouse nursery Himmerlands ApS, located in Storvorde, Denmark (56°56′06.7″N 10°06′27.4″E). At each nursery, the production of Christmas trees derived from the same parental seed source: Berritzgaard F 665 (providers: Levinsen A/S, Gørløse, Denmark). Entire plants from the field or the original Jiffy^®^ pots were removed with adjoining soil.

### Isolation of Root-Associated Bacteria From *A. nordmanniana* Plants

Entire root systems with firmly adhering soil were cut into short fragments and macerated in a mortar. A mix including rhizosphere soil and plant root tissue (endosphere plus rhizoplane) was thereby obtained and bacteria obtained from these samples are hereafter referred to as root-associated. Dilutions of the samples were spread uniformly on Luria-Bertani (LB) agar plates (Thermo-Fisher Scientific, Inc., Waltham, MA, United States) supplemented with Nystatin (50 mg L^−1^) (Sigma-Aldrich, Gillingham, United Kingdom) to avoid fungal growth. The plates were incubated for 48 h at 28°C and colony forming units (CFU) were determined. Colonies displaying variations in morphology were classified as different isolates, which were subsequently stored at −80°C in 30% glycerol.

### Bacterial DNA Extraction and Characterization of Isolates by Universally Primed PCR (UP-PCR) Fingerprinting

The selected isolates were grown overnight with shaking at 150 rpm in LB liquid medium (Sigma-Aldrich, Gillingham, United Kingdom) at 28°C. DNA from the bacterial isolates was released by boiling. For recovery of bacterial DNA, liquid cultures were washed twice in phosphate buffered saline (PBS) and subsequently re-suspended in sterile Milli-Q water. The re-suspended cells were incubated in a heating block at 99°C for 10 minutes and re-suspended into ice-cold sterile Milli-Q water. After centrifugation, the supernatants (bacterial lysates) were stored at −20°C until use. UP-PCR was performed for each of the isolates using the universal L15/AS19 primer (5′-GAGGGTGGCGGCTAG-3′) (Lübeck et al., 1998) following the procedures of Brandt et al. (2006). The resulting UP-PCR band patterns were grouped manually and isolates with the same band pattern were assigned to the same bacterial strain. Subsequently, one representative isolate for each strain was selected for taxonomic identification.

### Taxonomical Identification of Bacterial Strains by Partial 16S rRNA Gene Sequencing

Part of the bacterial 16S rRNA gene was amplified by PCR following the protocol of Garcia-Lemos et al. (2019); and using the primers 27 Forward (AGAGTTTGATCMTGGCTCAG), and 1492 Reverse (TACGGYTACCTTGTTACGACTT). Both primers were obtained from TAG Copenhagen A/S, Copenhagen, Denmark. The PCR amplification was conducted in a Mastercycler Pro thermal cycler (Eppendorf, Hauppauge, NY), as follows: an initial denaturation step consisting of 4 min at 94°C; 35 cycles of 30 s at 94°C, 1 min at 55°C, 1 min at 72°C, and a final extension of 7 min at 72°C.

After agarose gel electrophoresis the PCR products were purified using the QIAquick PCR Purification kit, following the manufacturer’s instructions (Qiagen Group, CA). Thereafter, the purified PCR products were sent to Eurofins Genomics (Eurofins Genomics, DE) for sequencing of the 16S rRNA gene region. The raw sequences received from Eurofins Genomics were edited using CLC Genomics Workbench Version 8.0^3^. The bacterial identification was based on 97–99% of similarities after matching the 16S rRNA gene sequences against the Greengenes nucleotide database using the Basic Local Alignment Search Tool (BLAST) (Altschul et al., 1990). The partial 16S rRNA DNA sequences of each identified isolate were deposited in the NCBI database under the project PRJNA515250.

### Seed Germination Trials in Germination Pouches

For the germination trials, 60 strains representing all the identified genera and species (Supplementary Table 1), were used as bacterial inoculants. The bacteria were grown in LB liquid medium for 48 h at 28°C with shaking at 150 rpm. Subsequently, the bacterial suspensions were adjusted to an Optical Density (OD) at 600 nm of 1, roughly corresponding to 10^9^ CFUs ml^–1^ (Zulueta-Rodríguez et al., 2015). Thereafter, four to five hundred seeds of *A. nordmanniana* Berritzgaard F 665 (Levinsen A/S) were surface sterilized by immersion in 0.5% sodium hypochlorite for 2 min at room temperature. The seeds were then rinsed three times in sterile distilled water and dried on sterile filter paper. The surface sterilized seeds of *A. nordmanniana* Berritzgaard F 665 were inoculated with the bacterial suspensions under orbital agitation at 150 rpm for 1 h at room temperature (Zulueta-Rodríguez et al., 2015). Then, the inoculated seeds, and the negative control performed with surface sterilized, “mock-inoculated” (with the used growth media without inoculants) seeds were transferred to sterile CYG^TM^ germination pouches (16.5 cm by 18 cm)^4^. Each seed germination pouch was placed in a growth chamber at 20°C, ∼40% relative humidity (RH), and a 12 hrs light/dark photoperiod. The germination pouches were inspected and re-watered every 2 days during a period of 20 days, and for the germination trial, six seeds per treatment with three replicates per treatment were used (for a total of 18 seeds per treatment), and with three independent repetitions (overall 54 seeds per treatment).

### Phytohormone Production by the Bacterial Strains

Phytohormone production was tested for bacterial pure cultures. For this, 50 μl of an overnight bacterial culture grown in LB medium, were transferred into a tube with 5 ml of LB medium supplemented (+) or not (−) with 200 μl of L-tryptophan (2 mg mL^−1^). The tubes were incubated in an orbital shaker at 28°C for 42 hrs. After the incubation, the tubes were centrifuged at 10,000 × *g* for 5 min, and the supernatant was collected and dried using a SpeedVac vacuum concentrator (Thermo Fisher Scientific, United Kingdom) at 43°C and 1000 rpm for approx. 8 hrs until the sample was completely dried. Samples were extracted according to Großkinsky et al. (2014) with modifications. Briefly, 1 ml of cold 80% methanol was added to each dried sample. Then, the samples were mixed using a vortex, and 4 μl of a mix of internal phytohormone standards (ISTD) were added to each sample, where after each sample was vortexed and incubated at 4°C for 30 min. After this, the samples were centrifuged at 10.000 × *g* for 15 min at 4°C, then the supernatants were passed through C18 columns (Chromafix, Macherey–Nagel, Düren/Germany) after a pre-equilibration with three times 1 ml 80% (v/v) methanol and flow-throughs were collected in tubes and kept on ice. Thereafter, the tubes were placed in a SpeedVac at 43°C until the samples were completely dried, and subsequently, the residues were dissolved in 1 ml 20% (v/v) methanol by brief sonication and filtered (MultiScreen HTS; EMD Millipore, cat no. MSGVN 2250). Phytohormones were analyzed by UHPLC/TQ-MS on an AdvanceTM-UHPLC/EVOQTMElite-TQ-MS instrument (Bruker, Madison, Wisconsin, United States) following the protocol of Martens et al. (2019). Three replicates per treatment (individual strains and a negative control of non-inoculated LB media with and without L-tryptophan) were analyzed.

### Effect of Bacterial Strains on Seed Germination and Growth of *A. nordmanniana* in the Greenhouse

For the greenhouse trials, *A. nordmanniana* seeds were inoculated following the same procedures as in the seed germination trials in germination pouches. Thereafter, inoculated seeds and control seeds were individually planted in plastic cone pots (3.8 cm diameter at the top and 20.32 cm deep) in non-sterile peat-based soil (pH: ∼4–4.5) (Miracle-Gro^®^ Sphagnum Peat Moss cat no. 85278430).

The greenhouse trial consisted of a complete randomized block design, with six blocks (trays with 96 pots in each). Each block included four treatments (3 bacterial strains + negative control), and for each treatment, 24 seeds were used per block for a total of 96 seeds (pots) per block and 576 seeds in total. Each block was placed randomly inside the greenhouse at day/night temperatures of 24°C/18°C, 40% relative humidity (RH), and the day length was extended to 16 hrs by Osram vialox Planta T lamps (400 W sodium HNT) for a minimum irradiance of 66 μmol quanta m^–2^ s^–1^ according to Ingvardsen et al. (2001). Plants were watered every 3 days with tap water to keep the soil moist and maintained for 30 days.

The impact of the three selected strains on plant growth and root development was determined after 1 month of growth. At the end of each experiment, the seedlings were removed carefully from the pots and the germination percentage was calculated. For subsequent analyses of plant growth and root development, some seedlings were washed, weighted, scanned, and analyzed using the software WinRHIZO^TM^ to measure the root length, the root volume, and the number of root tips. For subsequent assessment of bacterial inoculant persistence, seedlings were stored with adhering rhizosphere soil at −20°C prior to subsequent DNA extraction. Seedlings to be used for enzyme activity and carbohydrate measurements were separated into shoot, stem, and root tissue. Samples were stored at −80°C prior to subsequent analysis.

### Enzyme Activity Signatures of Plant Antioxidative Metabolism

The impact of bacterial strains on plant antioxidative activity signatures was tested by a semi-high throughput method to determine the activities of eight key enzymes of antioxidant metabolism in roots, stems, and shoots. For this, proteins were extracted from plant tissue samples according to Garcia-Lemos et al. (2019). Briefly, 100 mg ground material was mixed with 100 mg Amberlite XAD-4 and 100 mg PVPP and extracted using 1.5 ml of extraction buffer (100 mM potassium phosphate buffer, pH 7.0, 5 mM ascorbate, 5 mM Dithiothreitol (DTT), 5 mM sodium bisulfite, 7.5 mM MgCl_2_, 20 μM MnCl_2_, 10% glycerol, 1% Polyvinylpyrrolidone (PVP) (LaFever et al., 1994). Cell wall-bound proteins were extracted from the remaining pellet with a high-salt buffer (1 M NaCl, 40 mM TRIS–HCl, pH 7.6, 3 mM MgCl_2_, 1 mM EDTA, 0.1 mM Phenylmethylsulfonyl fluoride (PMSF), 1 mM benzamidine, 14 mM β-mercaptoethanol, 24 μM Nicotinamide adenine dinucleotide phosphate (NADP) according to Jammer et al. (2015).

Activities of the antioxidative enzymes ascorbate peroxidase (APX), catalase (CAT), cytoplasmic peroxidases (POX), glutathione reductase (GR), superoxide dismutase (SOD), glutathione S-transferase (GST), monodehydroascorbate reductase (MDHAR) and dehydroascorbate reductase (DHAR), were determined photometrically by kinetic assays in a miniaturized 96-well plate format following the approach presented in Jammer et al. (2015). The assays were based on principles published by Edwards et al. (1990); Polle et al. (1994), Yoshimura et al. (2000), and Garcia-Lemos et al. (2019). For each treatment, three biological replicates and three technical replicates were taken by each plant tissue used for analysis.

### Carbohydrate Determination in *A. nordmanniana* Samples

Soluble carbohydrates were extracted by 80% ethanol and subjected to Ion Chromatography (IC) analysis to determine sucrose (Suc), glucose (Glc) and fructose (Fru). Starch was determined after enzymatic hydrolysis of the remaining pellet and analyzed by IC. All analyses were performed on the integrated IC system 881 Compact IC pro (Metrohm Inula, Vienna). Detailed description of carbohydrate determination can be found in Supplementary Appendix 1.

Apart from the individual amounts of carbohydrates and starch determined, the following parameters were derived from these data: The hexose:sucrose ratio was determined, where hexose levels are represented by the sum of Glc and Fru. The sum of Glc, Fru and Suc served as an estimate of total soluble carbohydrates (Σ sol. CH). The sum of Glc, Fru, Suc and starch served as an estimate of non-structural carbohydrate amounts (Σ NSC).

### Colonization of *A. nordmanniana* by Bacterial Strains

To evaluate the rhizosphere colonization of the three bacterial strains used in the greenhouse experiment, a 16S rRNA gene amplicon sequencing approach was used. The loosely attached soil was removed from the roots by a brush and the roots with attached soil were macerated in a mortar with liquid nitrogen. The resulting samples are referred to as root-associated samples (including rhizosphere soil plus root tissue), and these samples represent TF (time final after 1 month of growth in the greenhouse). Additionally, six inoculated seeds per treatment representing T0, were used to extract DNA.

DNA from all samples (T0, TF and bulk soil samples), was extracted following the DNA extraction protocol of Paulin et al. (2013). The nucleic acid concentration of each extract was quantified using a NanoDrop 2000c spectrophotometer (Thermo Scientific, Wilmington, DE), (Desjardins and Conklin, 2010), and the samples were sent to BGI Co. Ltd. (BGI TECH SOLUTIONS, Hong Kong), for amplicon sequencing of the V3-V4 regions of the 16S rRNA gene using the primers 341F (ACTCCTACGGGAGGCAGCA) and 806R (GGACTACHVGGGTWTCTAATG) (Yang Y. et al., 2017). The sequencing generating paired-end reads was performed using a HiSeq 2500 PE300 Dual Index system.

Amplicon sequencing data was imported to CLC Genomics Workbench Version 8.0^5^. The raw data was processed following the CLC Microbial Genomics Module pipeline. Briefly, sequences were paired, whereafter the primer sequences were trimmed off following the procedures described in Garcia-Lemos et al. (2019). After that, the paired read sequences were merged, and the length was fixed by trimming to an exact length of 402 bp. Next, the samples were filtered to a minimum number of 100 reads per sample and the samples that did not fulfill this parameter were discarded. Afterward, an assignment to operational taxonomic units (OTUs) of the filtered sequences with comparable length and coverage was performed, by clustering the sequences and assigning the sequences to OTUs, based on 97% of similarity matched against the Greengenes 16S rRNA gene database. Finally, a list of sequences that clustered with sequences in the Greengenes database was used to generate the OTU abundance table. The OTU relative abundance table was generated by summing up all OTU counts and dividing each count with the total sum.

### Effect of Bacterial Strains on Growth Promotion of *A. nordmanniana* in the Field

Field trials were started in April 2017, where 300 uniform 2-year-old plants were divided into 4 groups that were subdivided into 3 replication groups of 25 plants. The entire root system of each plant was submerged in bacterial suspensions with 10^9^ CFUs ml^–1^ of either of the 3 selected strains or tap water for 24 h (control), and thereafter immediately planted in the field. Each replication group was planted according to a random block design with a distance between plants of 0.7 m in rows 2.0 m apart. To prevent weed growth, the topsoil of the rows was covered with Yuzet^®^ weed membranes. The plants were neither fertilized nor watered thereafter. The mineral composition in the field soil was determined by Eurofins Agro Testing Denmark^6^ and can be found in Supplementary Table 2. In November 2019, the plants were lifted. For each plant, the number of deeply penetrating roots and minor topsoil side roots were counted manually, and fresh weight was calculated using a calibrated weight. Furthermore, needles were selected from one top whorl branch and analyzed for the level of chlorophyll (a + b) as described by Veierskov and Ferguson (1991). Data are only presented for inoculation with the *Paenibacillus* sp. s37 strain (which showed the largest stimulation of root development under greenhouse conditions). The 16S rRNA gene amplicon sequences were deposited in NCBI’s Sequence Read Archive under BioProject PRJNA515250.

### Statistical Analysis

The data from laboratory, greenhouse and field experiments were tested for normality using Shapiro-Wilk W in PAST version (2.17) and subjected to one-way analysis of variance (ANOVA), to compare between treatments for seed germination, and growth promotion of *A. nordmanniana.* Then, a comparison between treatment’s means was performed using a *post hoc* Tukey’s Honestly Significant Difference (HDS) using R studio Version 1.1.463 (R. RStudio, Inc., Boston, MA^7^). Student’s *t*-test using PAST version (2.17), was performed to compare the differences in taxa abundance at genus level and filtering the *Bacillus* and *Paenibacillus* genera, between the samples at T0 (inoculated seeds before planted into pots), TF (rhizosphere samples after 1 month of growth at the greenhouse), negative control and soil samples. Significant differences in enzyme and carbohydrate levels in the shoot, stem and root were determined by a two-sided, unpaired Student’s *t*-test.

## Results

### Screening of Root-Associated Bacteria From *A. nordmanniana* Plants Collected at Field and Greenhouse Nurseries

**FIGURE 1:**
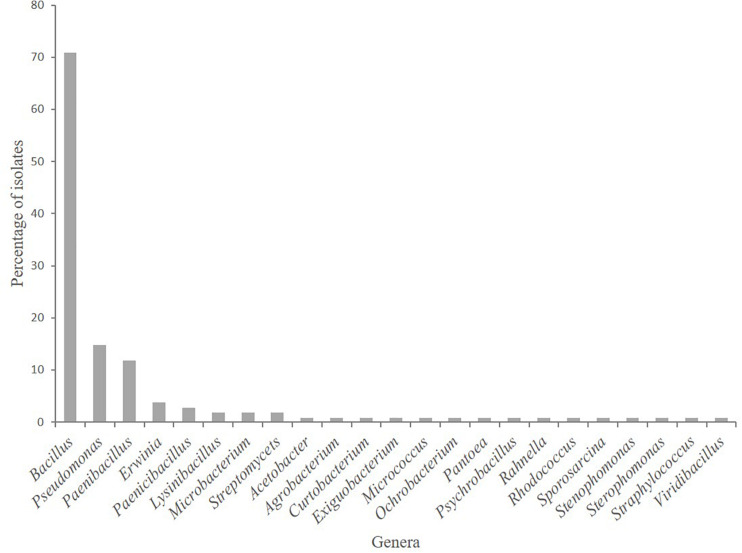
Root-associated bacterial strains isolated from field grown *Abies nordmanniana* plants. The relative abundance of strains at the genus level is presented (*n* = 126).

Five hundred fifty-one bacterial isolates, obtained from the rhizosphere soil plus root tissue (i.e., root-associated bacteria) of *A. nordmanniana* plants grown in the field or in the greenhouse nurseries, were assigned to 126 strains based on morphological characterization and UP-PCR fingerprinting (Supplementary Table 1). Based on sequencing of the 16S rRNA gene, 23 bacterial genera containing 40 bacterial species were represented in the strain collection. Members of the genus *Bacillus* were the most commonly isolated strains [particularly in the 3-year-old plants (Supplementary Figure S1)], accounting for 56% of the total number of strains, followed by *Pseudomonas* with 12%, and *Paenibacillus* with 9.5% (Figure 1).

Sixty bacterial strains representing the diversity of the strain collection were tested for their effect on *A. nordmanniana* seed germination in growth pouches (strains highlighted by blue in Supplementary Table 1). Of these stains, only 17 gave consistent results, and 12 significantly (*P* < 0.05) increased seed germination after 20 days of incubation (Supplementary Figure S2). Overall, *Paenibacillus* and *Bacillus* strains induced the highest germination percentages of above 40% (Supplementary Figure S2), with strains *Paenibacillus* sp. s37 and *Paenibacillus* sp. s40 showing the highest percentages of ca. 45%. Damage to the seeds by bacterial growth was not observed for any of the strains. The production of the phytohormone indole-3-acetic acid (IAA) was determined for the six strains giving the highest germination percentages. The screening showed that *Paenibacillus* sp. s12, *Paenibacillus* sp. s37, *Paenibacillus* sp. s40 and *Bacillus* sp. s50, all produced IAA and that production was highest in the presence of L-tryptophan with 244 pmol g FW^–1^ for *Paenibacillus* sp. s37, 125 pmol g FW^–1^ for *Bacillus* sp. s50, and 112 pmol g FW^–1^ for *Paenibacillus* sp. s40 (Supplementary Table 3).

### Effect of Selected Bacterial Strains on Seed Germination and Growth of *A. nordmanniana* in Greenhouse Trials

**FIGURE 2:**
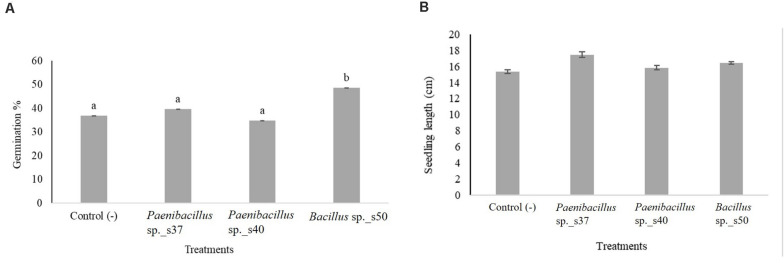
The effect of selected bacterial strains on seed germination and growth of *A. nordmanniana* under greenhouse conditions. **(A)** Germination percentage; and **(B)** Seedling length of *A. nordmanniana* after 30 days of growth. Seeds were inoculated with cell suspensions of the three bacteria showing the highest seed germination in growth pouches. Non-inoculated seeds served as the negative control. The figure shows the mean values from three independent repetitions, each including six replicates. Different letters indicate significantly different means at *P* < 0.05 (*Post hoc* test). Each bar represents the Standard Error.

The three bacterial isolates yielding the highest germination percentages and auxin production in the laboratory (i.e., *Bacillus* sp. s50, *Paenibacillus* sp. s37 and *Paenibacillus* sp. s40) were tested in a greenhouse experiment where *A. nordmanniana* seeds were planted in soil. Inoculation with *Bacillus* sp. s50 resulted in a significantly (*P* < 0.05) higher seed germination percentage (48.6%) in these trials than the non-inoculated control (36.8%), while the two remaining strains did not significantly stimulate seed germination in the greenhouse (Figure 2A).

**FIGURE 3:**
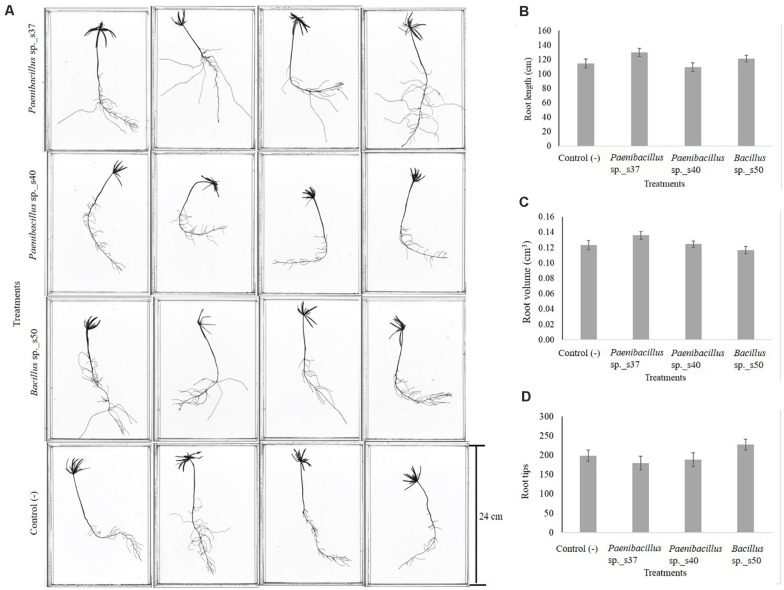
Effect of selected root-associated bacterial strains on growth of *A. nordmanniana* under greenhouse conditions. Seeds were inoculated with bacterial cell suspensions and sowed pots with non-sterilized soil, which were kept at the greenhouse at 18°C for 30 days. After 30 days of growth, the seedlings were scanned **(A)** and measured for root length (cm) **(B)**, root volume (cm^3^) **(C)**, and number of root tips **(D)**. The figure shows the average values of six replicates per treatment. Each bar represents the Standard Error.

Seed inoculation with any of the three strains led to increased seedling lengths, but the differences were not significant compared to the control (Figure 2B). Visual inspection revealed that seedlings developing from the seeds inoculated with *Paenibacillus* sp. s37 or *Bacillus* sp. s50 showed longer secondary roots compared with the control (Figure 3A). However, the analyzed root scans did not show statistically significant differences between the treatments for root length (Figure 3B), root volume (Figure 3C), and number of root tips (Figure 3D). Never the less, a slight increase in the root length (130 cm versus control value of 114 cm) and root volume (0.14 cm^3^ versus 0.12 cm^3^) observed for seedlings treated with *Paenibacillus* sp. s37 (Figure 3B) corresponded with a small decrease in the number of root tips (179 versus 199) observed for the greenhouse seedlings (in accordance with the fewer and longer side roots observed visually).

### Antioxidative Enzyme Activities in Greenhouse Trials

Overall, the three bacterial strains showed clear and distinct effects on the antioxidative enzyme activity level in the different plant tissues (Table 1). For *Paenibacillus* sp. s37, the inoculated plants showed a significant (*P* = 0.05) increase of the MDHAR activity in root tissue compared to control plants (0.80 versus 0.49 nkat g FW^–1^). Moreover, in stem tissue, these plants showed a significant (*P* = 0.03) decrease of the DHAR activity (9.06 versus 23.03 nkat g FW^–1^). Finally, for shoot tissue, *Paenibacillus* sp. s37 inoculated plants showed a significant (*P* = 0.004) decrease of the GST activity (8.52 versus 14.10 nkat g FW^–1^ (Table 1).

**TABLE 1:** Activity profile of eight antioxidant enzymatic signature in the different plant tissues of *A. nordmanniana* seedlings from seeds inoculated with selected bacterial strains and non-inoculated seeds (Control −) grown under non-stressed greenhouse conditions.

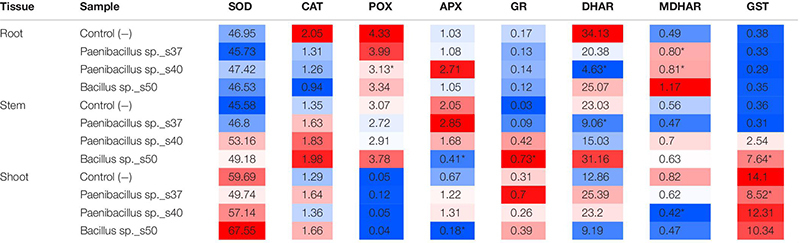

In addition to numeric means data are represented as a heatmap based on a gradient red-white-blue color scale. Dark red color indicates highest, dark blue color lowest values for each enzyme. Data are presented as mean values [nkat g FW^−1^] derived from three biological replicates, each including three technical replicates (*n* = 9). * Significantly different to control (*P* < 0.05), based on two-sided, unpaired Student’s *t*-test. Superoxide dismutase (SOD), Catalase (CAT), Peroxidase (POX), Ascorbate peroxidase (APX), Glutathione reductase (GR), Dehydroascorbate reductase (DHAR), Monodehydroascorbate reductase activity (MDHAR), Glutathione S-transferase (GST).

Plants inoculated with *Paenibacillus* sp. s40 showed a significant (*P* = 0.01) decrease in the activities of POX and DHAR in root tissue (from 4.33 to 3.13 nkat g FW^–1^ and 34.13 to 4.63 nkat g FW^–1^, respectively), while a significant increase was seen for the MDHAR activity (*P* = 0.04) with 0.81 nkat g FW^–1^ compared to control plants with 0.49 nkat g FW^–1^. For shoot tissue a significant (*P* = 0.04) decrease of the MDHAR activity from 0.82 to 0.42 nkat g FW^–1^ was observed in plants inoculated with *Paenibacillus* sp. s40 (Table 1).

Finally, plants inoculated with *Bacillus* sp. s50 showed a significant (*P* = 0.02) decrease of the APX activity from 2.05 to 0.41 nkat g FW^–1^ in stem tissue, while the same treatment showed significant increases (*P* = 0.01) in the GR activity (from 0.03 to 0.73 nkat g FW^–1^) and the GST activity (*P* = 0.005) (from 0.36 to 7.64 nkat g FW^–1^) in this tissue (Table 1). For shoot tissue, strain s50 caused a significant (*P* < 0.01) decrease in the APX activity from 0.67 to 0.18 nkat g FW^–1^.

### Plant Carbohydrate Levels in the Greenhouse Trials

The highest individual and total soluble carbohydrate levels (Glc, Fru, Suc and Σsol. CH) were found in shoot samples (Table 2). Additionally, higher hexose and Σsol. CH levels was found in stem compared to root samples, whereas the opposite was seen for sucrose (Table 2). The ratio of free hexose levels to levels of the transport sugar sucrose (Hex/Suc) was always highest in stem and lowest in shoot samples.

**TABLE 2:** Carbohydrate levels in the different plant tissues of *A. nordmanniana* seedlings from plants inoculated with selected bacterial strains and non-inoculated seeds [Control (−)] grown under non-stressed greenhouse conditions.

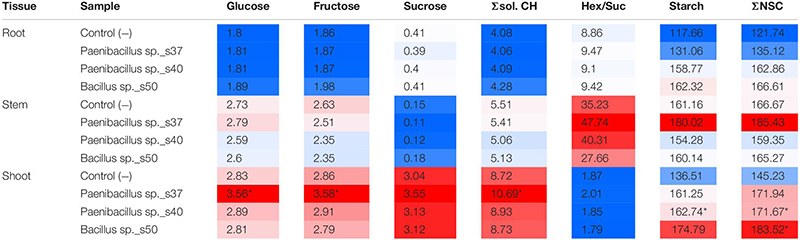

In addition to numeric means, data are represented as a heatmap based on a gradient red-white-blue color scale. Dark red color indicates highest, dark blue color lowest values for each carbohydrate. Data are presented as mean values [mg g FW^−1^] derived from three biological replicates, each including three technical replicates (*n* = 9). * Significantly different to control (*P* < 0.05), based on two-sided, unpaired Student’s *t*-test. Σsol. CH, estimate of total soluble carbohydrates; Hex/Suc, hexose:sucrose ratio; ΣNSC, estimate of non-structural carbohydrate amounts.

Overall, *Paenibacillus* sp. s37 had the strongest and most consistent impact on plant carbohydrate levels. *Paenibacillus* sp. s37 treatment resulted in significantly (*P* < 0.01) higher levels of Glc (3.56 mg g FW^–1^), Fru (3.58 mg g FW^–1^) and Σsol. CH (10.69 mg g FW^–1^) than in the control for shoot tissues. Control values were 2.83 mg g FW^–1^, 2.86 mg g FW^–1^ and 0.72 mg g FW^–1^, respectively. In contrast the levels of these carbohydrates were not significantly affected in roots and stems (Table 2). Moreover, inoculation with this strain resulted in increased accumulation (significant or by trend) of all carbohydrate parameters in shoots and increased starch and total non-structural carbohydrates ΣNSC in all tissues compared to the controls (Table 2). Furthermore, the levels of ΣNSC were also significantly (*P* < 0.05) increased in *Paenibacillus* sp. s40- and *Bacillus* sp. s50-treated seedlings, which showed ΣNSC values of 171.67 mg g FW^–1^ and 183.52 mg g FW^–1^, respectively, for the shoot tissue versus the control value of 145.23 mg g FW^–1^. A corresponding increase to 171.94 mg g FW^−1^ for *Paenibacillus* sp. s37-treated seedlings was not significant (Table 2).

### Persistence of Bacterial Strains in the Greenhouse Trials

**FIGURE 4:**
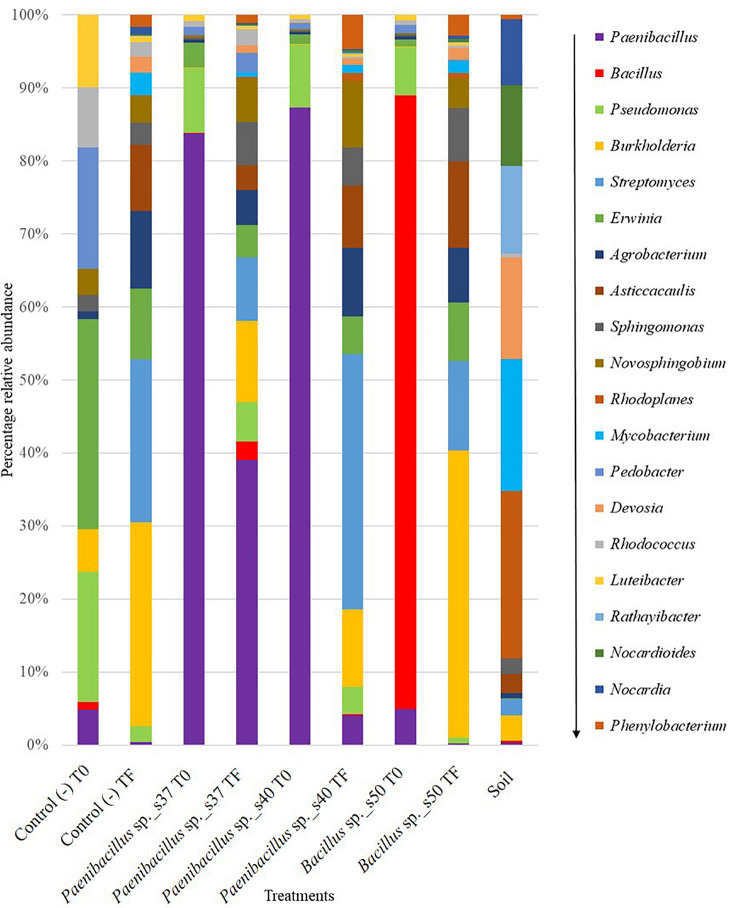
Stacked bar plot showing the relative abundance of bacterial OTUs classified to the genus level (only top 20 genera with the highest relative abundance are shown) in all treatments before (T0 = the seed at inoculation) and after (TF = the roots after 30 days) the greenhouse growth period. Data are based on the 16S rRNA amplicon sequencing of three biological replicates per sample. The panel at the right shows the bacterial genera in descending order from the genus with the highest relative abundance to the genus with lower relative abundances across all samples.

From the amplicon sequencing analysis targeting the 16S rRNA genes of bacterial communities, the relative abundance of the taxa (Genera) including the three inoculated strains was determined for the inoculated seed (T0) and for the root samples, i.e., rhizosphere soil plus root tissue, after 1 month of growth (TF). The relative abundances were then used as a proxy for bacterial persistence in the rhizosphere soil and their possible endophytic root colonization in the experiment. Overall, in the non-inoculated seeds (Control (−) T0), the most abundant genera were: *Erwinia, Pseudomonas, Pedobacter* and *Luteibacter*, all with 10% or more of relative abundance (Figure 4). Additionally, in the root samples of control plants after 1 month of growth (Control (−) TF), the most abundant genera were: *Erwinia, Burkholderia* and *Streptomyces* with 10% or more of relative abundance (Figure 4).

The 16S rRNA-targeted analysis confirmed a high relative abundance of the genus *Paenibacillus* for seeds inoculated with *Paenibacillus* sp. s37 (83%) or *Paenibacillus* sp. s40, (87%) (T0). For the root samples after 1 month of growth in the greenhouse (TF), *Paenibacillus* was found in significantly (*P* < 0.05) higher relative abundance (39%) in plants inoculated with *Paenibacillus* sp. s37, than in plants inoculated with *Paenibacillus* sp. s40 (4%), *Bacillus* sp. s50 (0.3%), or in non-inoculated controls (0.5%) (Figure 4).

For *Bacillus*, the sequencing analysis confirmed a significantly (*P* < 0.0001) higher relative abundance of this genus for the seeds inoculated with *Bacillus* sp. s50 (84%) at (T0) (Figure 4). However, in the root samples after 1 month of growth (TF), the relative abundance of *Bacillus* was significantly (*P* < 0.001) lower than at T0 of the same treatment and not significantly different (*P* > 0.05) from their relative abundance in the non-inoculated control. In the seed (T0) and root (TF) samples from the negative controls, as well as in the soil samples, the relative abundance of *Paenibacillus* and *Bacillus* was less than 2% of the total bacterial community OTUs (Figure 4).

### Effects of *Paenibacillus* sp. s37 in the Field

After two growth seasons in the field (18 months), the plants were lifted, and the root system evaluated. Plants treated with *Paenibacillus* sp. s37 had nearly doubled the number of secondary roots (*P* < 0.05), whereas the number of large tap roots was unaffected by the treatment, yielding a dense root structure in the topsoil layer (Table 3 and Supplementary Figure S3). At the same time, the level of chlorophyll (a + b) increased significantly (*P* < 0.05) by 12% in the needles of the first whorl compared to the level in control plants (Table 3).

**TABLE 3:** Effect of seed inoculation with isolated root-associated bacterial strain *Paenibacillus* sp. s37 on root development and chlorophyll levels of *A. nordmanniana* after 2 years of growth under field conditions.

Treatment	Control (−)	*Paenibacillus* sp._s37
Number of main tap roots	4.72.2	4.51.8
Number of secondary roots	23.46.7	43.84.3*
FW (g) secondary roots	23.85.9	48.37.7*
Chl (a + b) (mg/g) of first whorl needles	1.040.02	1.170.04*

Numbers represent mean values of six replicates per treatment. * Significant differences (*P* < 0.05); FW, fresh weight; Chl, chlorophyll.

## Discussion

The current screening for root-associated PGPR strains took off from a collection of strains yielding good seed germination and auxin production under laboratory conditions, as plant growth promoting effects of many PGPRs are assigned to bacterial production of phytohormones such as IAA (Bais et al., 2006; Sasse et al., 2018; Berger et al., 2020). The strain collection was dominated by strains belonging to the genera *Bacillus, Pseudomonas* and *Paenibacillus*, which contain many reported PGPR (Idris et al., 2004; Kour et al., 2019; Shelake et al., 2019). Interestingly, *Bacillus*, *Pseudomonas* and *Paenibacillus* were not very abundant in the *A. nordmanniana* root samples according to the current 16S rRNA gene-targeted sequencing analysis as well as data from Garcia-Lemos et al. (2019, 2020). This confirms a cultivation bias that leads to over-representation of Firmicutes and Proteobacteria in cultivation-dependent surveys as discussed by Overmann et al. (2017). The screening pipeline included laboratory, greenhouse and field experiments as we wanted to include conditions relevant for the growers (i.e., seed germination in greenhouses and growth in both greenhouses and the field). We recovered two strains that significantly improved different aspects of plant growth and hence confirmed the hypothesis set out for this study.

*Bacillus* sp. s50 caused a significant promotion of seed germination in *A. nordmanniana* under greenhouse conditions and changed root morphology toward fewer, long lateral roots. The positive effect on seed germination is in line with a study by Wagner et al. (2019) showing that a *Bacillus* sp. strain produced IAA and stimulated the seed germination and shoot growth in *Picea abies* grown in hydroponic systems. A role of auxins in conifer seed germination has further been supported by promotion of seed germination by exogenously applied IAA in *Pinus massoniana* (Guangwu and Xuwen, 2014). However, the role of IAA on seed germination has been debated as this hormone also controls seed dormancy in other plants (Liu et al., 2013). Concerning the effect of strain s50 on root morphology, the current results are in line with previous studies on production of IAA by *Bacillus* and *Paenibacillus*, and the important role of this phytohormone in shaping plant root architecture (Pindi et al., 2014; Grady et al., 2016; Dahmani et al., 2020).

*Bacillus* sp. s50 did not affect seedling length, root length or root biomass, nor did it increase the level of soluble hexoses, which could promote plant growth by serving as energy sources, signaling compounds, and building blocks (Sakr et al., 2018). In contrast the s50-mediated increase in total non-structural carbohydrates, in particular starch, points to an increase in carbon storage (Magel et al., 2000).

A significant impact of *Bacillus* sp. s50 on plant antioxidative enzyme activity profiles was seen as increased GR and GST activities but decreased APX activity. Hence, the bacterium did not affect the total ascorbate-glutathione cycle, which is important for scavenging H_2_O_2_ and providing reduced forms of the antioxidants ascorbate and glutathione (Hernández et al., 2017). While GR activity is induced by several abiotic stressors (Caverzan et al., 2016), increased GR as well as GST activities, which lead to an induced systemic resistance response, have previously been reported during interactions between plants and beneficial bacteria (Hernández et al., 2017; Gullner et al., 2018). Hence, it would be of interest to determine if *Bacillus* s50 induces systemic resistance in *A. nordmanniana* as such induction would improve the utility of this strain as a plant beneficial inoculant.

Across greenhouse and field experiments, *Paenibacillus* sp. s37 stimulated, significantly or by trend, several aspects of plant growth including seedling length, root length and the formation of secondary roots. These results are in agreement with recent literature, as many *Bacillus* and *Paenibacillus* strains have been reported to induce root and shoot elongation in distinct conifer species (Shishido et al., 1996; Zulueta-Rodríguez et al., 2015; Wagner et al., 2019). An involvement of IAA production by *Paenibacillus* sp. s37 was indicated by the increase of long lateral roots and the role of IAA in shaping plant root architecture as discussed above (Pindi et al., 2014; Grady et al., 2016; Dahmani et al., 2020). Moreover, IAA producing *Bacillus* and *Paenibacillus* strains have been reported to stimulate shoot growth of *Picea alba* (Wagner et al., 2019).

We speculate that the stronger effect of *Paenibacillus* sp. s37 on root development in the long-term field experiment than in 30-days greenhouse experiments might be due to the natural slow growth of conifers. This notion is supported by the studies of Shishido et al. (1996) and Zulueta-Rodríguez et al. (2015) who documented significant effects of bacterial inoculants (*Bacillus* spp. and *Pseudomonas* spp.) on seedling growth and root biomass of *Pseudotsuga menziesii*, *Abies hickelii* and *A. religiosa* after 3 and 4 months under greenhouse conditions, respectively. Hence, long-term experiments are required to consolidate a significant effect of bacterial inoculants on growth promotion, even for *A. nordmanniana.*

In line with the growth promotion, *Paenibacillus* sp. s37 stimulated several growth-associated physiological parameters. Hence, seedlings inoculated with *Paenibacillus* sp. s37 consistently showed an increased hexose:sucrose ratio in all tissues analyzed. This finding could be indicative for an increased liberation of hexoses from sucrose driven by the presence of *Paenibacillus* sp. s37 and the hexoses could promote plant growth by serving as energy sources or building blocks (Sakr et al., 2018). Moreover, *Paenibacillus* sp. s37 increased starch and non-structural carbohydrate (NSC) levels in all tissues, even though not significantly. Hence, these results document the ability of *Paenibacillus* strain s37 to enhance the concentrations of both free and storage carbohydrates and affect their allocation in the different plant tissues. Other bacterial inoculants have been reported to increase affect plant carbohydrate levels (Marcos et al., 2016; Qin et al., 2016) but the mechanism behind the effect of bacterial inoculations on the concentrations of plant carbohydrates is still poorly understood (Magel et al., 2000; Gagné-Bourque et al., 2016). In consequence, there is a need to implement physiological phenotyping into a holistic phenomics approach (Großkinsky et al., 2015, 2018) to clarify how bacterial inoculation affects the regulation of carbohydrate metabolism in *A. nordmanniana.*

The increased chlorophyll level (greenness) in the needles after inoculation with *Paenibacillus* sp. s37 is in agreement with reports showing that other bacterial inoculants can increase chlorophyll levels (Xie et al., 2009; Aghai et al., 2019) and the photosynthetic rate in other plants (Xie et al., 2009). It would be interesting if future studies on strain s37 could link the impact on growth with the effects on photosynthesis and on carbohydrate levels in shoot tissue, to determine if increased photosynthesis leads to improved vigor via carbohydrate accumulation. Finally, *Paenibacillus* strain s37 promoted plant growth with a minor impact on plant antioxidative enzyme profiles suggesting a limited potential for mitigation of biotic or abiotic stress via priming the antioxidant system. Likewise, the growth promotion of maize by *Bacillus licheniformis* FMCH 001 was found to have only a limited impact on antioxidant metabolism (Akhtar et al., 2020).

The root-associated bacterial community of *A. nordmanniana* was dominated by taxa (i.e., *Erwinia, Burkholderia* and *Streptomyces*), which have previously been observed by comparable analysis in non-inoculated *A. nordmanniana* plants from several geographical locations (Garcia-Lemos et al., 2019, 2020). In addition, our first data for the seed-associated bacterial communities of *A. nordmanniana* documents dominating taxa (*Erwinia, Pseudomonas, Pedobacter* and *Luteibacter*) affiliated with the Proteobacteria and Bacteroidetes, which are abundant in seed-associated bacterial communities across many plant species (Barret et al., 2015; Nelson, 2018; Rodríguez et al., 2018). For comparison, seeds of Norway spruce harbor *Pseudomonas* and *Rahnella* living as endophytes (Cankar et al., 2005) as shown by cultivation-dependent analysis, while we have not been able to find information on bacterial communities in conifer seeds obtained by 16S rRNA-targeted community analysis.

The relative abundance of the genus *Paenibacillus* spp. in *A. nordmanniana* roots was maintained at a high level as compared to the non-inoculated control for 1 month after inoculation with *Paenibacillus* sp. s37. Although our analysis includes native *Paenibacillus* populations, the data suggest good persistence in the rhizosphere under close to natural conditions. Strain s37 might even colonize the endosphere and establish as an endophyte, as the root tissues were also included for bacterial DNA extraction. However, additional experiments distinguishing between root compartments (rhizosphere soil, rhizoplane and endosphere), or colonization assays using a *gfp*-tagged strain will be required to determine the specific niche colonized by *Paenibacillus* sp. s37. *Paenibacillus* spp. have previously been reported as endophytes. For example, *P. polymyxa* can consistently colonize the endosphere when inoculated in diverse plant hosts as lodgepole pine, and its ability to degrade major cell wall components might facilitate the colonization of internal root tissues (Yang H. et al., 2017).

In contrast, *Bacillus* sp. strain s50 did not maintain a high root-associated population in the greenhouse experiments. For comparison, the abundance of *Bacillus velezensis* (formerly known as *B. amyloliquefaciens*) biocontrol strain FZB42 in lettuce rhizosphere gradually decreased during 6 weeks of plant growth. Nevertheless, FZB42 reduced disease caused by plant-borne fungal pathogens (Chowdhury et al., 2015) indicating that relative few *Bacillus* cells can exert a plant beneficial function. Such behavior would be compatible with a potential induction of plant resistance by s50 early after inoculation as discussed above.

*A. nordmanniana* is characterized by slow growth during the first 3 years in the nurseries. Improvements in seed germination, root development, which facilitate increased nutrient uptake in the plant, and growth of the seedlings are critical for increased survival and establishment of this species after the nursery stage (Seifert, 2015). With the current greenhouse and field trials, we were able to document that the selected strains stimulated seed germination or showed plant growth promoting traits under conditions that are used in Christmas tree nurseries. Our results also suggest that the bacterial strains *Bacillus* sp. s50 and *Paenibacillus* sp. s37 could supplement each other by showing promising results in seed germination and root development, respectively. Further studies are required to decipher in detail the mechanisms, by which the selected strains enhance seed germination and growth of *A. nordmanniana*, aiming to explore their PGP potential also under stressful conditions that might harm the growth of this tree crop under the current changing climatic conditions.

## Data Availability Statement

The datasets presented in this study can be found in online repositories. The names of the repository/repositories and accession number(s) can be found in the article/ Supplementary Material.

## Author Contributions

AG-L, ON, MN, TR, and BV contributed to the experimental design. AG-L, ON, and BV contributed to the sampling. AG-L, DKG, SS, and BV carried out the experiments. All authors contributed to the acquisition and analysis of the data, writing, and editing of the manuscript and approved the final version.

## Conflict of Interest

The authors declare that the research was conducted in the absence of any commercial or financial relationships that could be construed as a potential conflict of interest.

## Footnotes

1 http://www.ctgce.com

2 https://www.christmastree.dk

3 https://www.qiagenbioinformatics.com/

4 https://mega-international.com/

5 https://www.qiagenbioinformatics.com/

6 www.eurofins.dk

7 http://www.rstudio.com/

## References

[B1] Aghai M. M. Khan Z. Joseph M. R. Stoda A. M. Sher A. W. Ettl G. J. (2019). The effect of microbial endophyte consortia on *Pseudotsuga menziesii* and *Thuja plicata* survival, growth, and physiology across edaphic gradients. *Front. Microbiol.* 10:1353. 10.3389/fmicb.2019.01353 31275276 PMC6591459

[B2] Akhtar S. S. Amby D. B. Hegelund J. N. Fimognari L. Großkinsky D. K. Westergaard J. C. (2020). *Bacillus licheniformis* FMCH001 increases water use efficiency via growth stimulation in both normal and drought conditions. *Front. Plant Sci.* 11:297. 10.3389/fpls.2020.00297 32318078 PMC7155768

[B3] Altschul S. F. Gish W. Miller W. Myers E. W. Lipman D. J. (1990). Basic local alignment search tool. *J. Mol. Biol.* 215 403–410. 10.1016/S0022-2836(05)80360-2 2231712

[B4] Bais H. P. Weir T. L. Perry L. G. Gilroy S. Vivanco J. M. (2006). The role of root exudates in rhizosphere interactions with plants and other organisms. *Annu. Rev. Plant Biol.* 57 233–266. 10.1146/annurev.arplant.57.032905.105159 16669762

[B5] Bakker P. A. H. M. Doornbos R. F. Zamioudis C. Berendsen R. L. Pieterse C. M. J. (2013). Induced systemic resistance and the rhizosphere microbiome. *Plant Pathol. J.* 29 136–143. 10.5423/PPJ.SI.07.2012.0111 25288940 PMC4174772

[B6] Bal A. Chanway C. P. (2012). Evidence of nitrogen fixation in lodgepole pine inoculated with diazotrophic *Paenibacillus polymyxa*. *Botany* 90 891–896. 10.1139/B2012-044

[B7] Barret M. Briand M. Bonneau S. Préveaux A. Valière S. Bouchez O. (2015). Emergence shapes the structure of the seed microbiota. *Appl. Environ. Microbiol.* 81 1257–1266. 10.1128/AEM.03722-14 25501471 PMC4309697

[B8] Berger S. Van Wees S. C. M. Nybroe O. Großkinsky D. K. (2020). Cross-frontier communication: phytohormone functions at the plant-microbe interface and beyond. *Front. Plant Sci.* 11:386. 10.3389/fpls.2020.00386 32322260 PMC7156614

[B9] Bharti N. Pandey S. S. Barnawal D. Patel V. K. Kalra A. (2016). Plant growth promoting rhizobacteria *Dietzia natronolimnaea* modulates the expression of stress responsive genes providing protection of wheat from salinity stress. *Sci. Rep.* 6:34768. 10.1038/srep34768 27708387 PMC5052518

[B10] Brandt K. K. Petersen A. Holm P. E. Nybroe O. (2006). Decreased abundance and diversity of culturable *Pseudomonas* spp. populations with increasing copper exposure in the sugar beet rhizosphere. *FEMS Microbiol. Ecol.* 56 281–291. 10.1111/j.1574-6941.2006.00081.x 16629757

[B11] Caverzan A. Casassola A. Brammer S. P. (2016). Antioxidant responses of wheat plants under stress. *Genet. Mol. Biol.* 39 1–6. 10.1590/1678-4685-GMB-2015-0109 27007891 PMC4807390

[B12] Cankar K. Kraigher H. Ravnikar M. Rupnik M. (2005). Bacterial endophytes from seeds of Norway spruce (*Picea abies L.* Karst), *FEMS Microbiol. Lett.* 244, 341–345. 10.1016/j.femsle.2005.02.008 15766788

[B13] Chowdhury S. P. Hartmann A. Gao X. Borriss R. (2015). Biocontrol mechanism by root-associated *Bacillus amyloliquefaciens* FZB42 - a review. *Front. Microbiol.* 6:780. 10.3389/fmicb.2015.00780 26284057 PMC4517070

[B14] Dahmani M. A. Desrut A. Moumen B. Verdon J. Mermouri L. Kacem M. (2020). Unearthing the plant growth promoting traits of *Bacillus megaterium* rmbm31, an endophytic bacterium isolated from root nodules of *Retama monosperma*. *Front. Plant Sci.* 11:124 10.3389/fpls.2020.00124 PMC7055178 32174934

[B15] Das P. Nutan K. K. Singla-Pareek S. L. Pareek A. (2015). Oxidative environment and redox homeostasis in plants: dissecting out significant contribution of major cellular organelles. *Front. Environ. Sci.* 2:70 10.3389/fenvs.2014.00070

[B16] Desjardins P. Conklin D. (2010). NanoDrop microvolume quantitation of nucleic acids. *J. Vis. Exp.* 45:e2565. 10.3791/2565 21189466 PMC3346308

[B17] Edwards E. A. Rawsthorne S. Mullineaux P. M. (1990). Subcellular distribution of multiple forms of glutathione reductase in leaves of pea (*Pisum sativum* L.). *Planta* 180 278–284. 10.1007/BF00194008 24201957

[B18] El-Lithy M. E. Reymond M. Stich B. Koornneef M. Vreugdenhil D. (2010). Relation among plant growth, carbohydrates and flowering time in the *Arabidopsis* Landsberg *erecta* × Kondara recombinant inbred line population. *Plant Cell Environ.* 33 1369–1382. 10.1111/j.1365-3040.2010.02155.x 20374533

[B19] Etesami H. Alikhani H. A. Hosseini H. M. (2015). Indole-3-acetic acid (IAA) production trait, a useful screening to select endophytic and rhizosphere competent bacteria for rice growth promoting agents. *MethodsX* 2 72–78. 10.1016/j.mex.2015.02.008 26150974 PMC4487705

[B20] Fimognari L. Dölker R. Kaselyte G. Jensen C. N. G. Akhtar S. S. Großkinsky D. K. (2020). Simple semi-high throughput determination of activity signatures of key antioxidant enzymes for physiological phenotyping. *Plant Methods* 16:42 10.1186/s13007-020-00583-8 PMC7085164 32206082

[B21] Gagné-Bourque F. Bertrand A. Claessens A. Aliferis K. A. Jabaji S. (2016). Alleviation of drought stress and metabolic changes in timothy (*Phleum pratense* L.) colonized with *Bacillus subtilis* B26. *Front. Plant Sci.* 7:584. 10.3389/fpls.2016.00584 27200057 PMC4854170

[B22] Garcia-Lemos A. M. Gobbi A. Nicolaisen M. H. Hansen L. H. Roitsch T. Veierskov B. (2020). Under the Christmas tree: belowground bacterial associations with *Abies nordmanniana*, across production systems and plant development. *Front. Microbiol.* 11:198. 10.3389/FMICB.2020.00198 32194515 PMC7064441

[B23] Garcia-Lemos A. M. Großkinsky D. K. Stokholm M. S. Lund O. S. Nicolaisen M. H. Roitsch T. G. (2019). Root-associated microbial communities of *Abies nordmanniana*: insights into interactions of microbial communities with antioxidative enzymes and plant growth. *Front. Microbiol.* 10:1937. 10.3389/fmicb.2019.01937 31507556 PMC6714061

[B24] Ghorbanpour M. Hatami M. Khavazi K. (2013). Role of plant growth promoting rhizobacteria on antioxidant enzyme activities and tropane alkaloid production of *Hyoscyamus niger* under water deficit stress. *Turk. J. Biol.* 37 350–360. 10.3906/biy-1209-12 31411186

[B25] Glick B. R. (2012). Plant growth-promoting bacteria: mechanisms and applications. *Scientifica* 2012:963401. 10.6064/2012/963401 24278762 PMC3820493

[B26] Govindasamy V. Senthilkumar M. Magheshwaran V. Kumar U. Bose P. Sharma V. (2010). *Bacillus* and *Paenibacillus* spp.: potential PGPR for sustainable agriculture. *Microbiol. Monogr.* 18 333–364. 10.1007/978-3-642-13612-2_15

[B27] Grady E. N. MacDonald J. Liu L. Richman A. Yuan Z. C. (2016). Current knowledge and perspectives of *Paenibacillus*: a review. *Microb. Cell Fact.* 15:203. 10.1186/s12934-016-0603-7 27905924 PMC5134293

[B28] Großkinsky D. K. Tafner R. Moreno M. V. Stenglein S. A. García de Salamone I. E. Nelson L. M. (2016). Cytokinin production by *Pseudomonas fluorescens* G20-18 determines biocontrol activity against *Pseudomonas syringae* in *Arabidopsis*. *Sci. Rep.* 6:23310. 10.1038/srep23310 26984671 PMC4794740

[B29] Großkinsky D. K. Albacete A. Jammer A. Krbez P. van der Graaff E. Pfeifhofer H. (2014). A rapid phytohormone and phytoalexin screening method for physiological phenotyping. *Mol. Plant* 7 1053–1056. 10.1093/mp/ssu015 24503160

[B30] Großkinsky D. K. Svensgaard J. Christensen S. Roitsch T. (2015). Plant phenomics and the need for physiological phenotyping across scales to narrow the genotype-to-phenotype knowledge gap. *J. Exp. Bot.* 66 5429–5440. 10.1093/jxb/erv345 26163702

[B31] Großkinsky D. K. Syaifullah S. J. Roitsch T. (2018). Integration of multiomics techniques and physiological phenotyping within a holistic phenomics approach to study senescence in model and crop plants. *J. Exp. Bot.* 69 825–844. 10.1093/jxb/erx333 29444308

[B32] Guangwu Z. Xuwen J. (2014). Roles of gibberellin and auxin in promoting seed germination and seedling vigor in *Pinus massoniana*. *For. Sci.* 60 367–373. 10.5849/forsci.12-143

[B33] Gullner G. Komives T. Király L. Schröder P. (2018). Glutathione S-transferase enzymes in plant-pathogen interactions. *Front. Plant Sci.* 9:1836. 10.3389/fpls.2018.01836 30622544 PMC6308375

[B34] Helepciuc F. E. Mitoi M. E. Manole-Pǎunescu A. Aldea F. Brezeanu A. Cornea C. P. (2014). Induction of plant antioxidant system by interaction with beneficial and/or pathogenic microorganisms. *Rom. Biotechnol. Lett.* 19 9366–9375.

[B35] Hernández J. A. Barba-Espín G. Diaz-Vivancos P. (2017). “Glutathione-mediated biotic stress tolerance in plants,” in *Glutathione in Plant Growth, Development, and Stress Tolerance*, eds Hossain M. Mostofa M. Diaz-Vivancos P. Burritt D. Fujita M. Tran L. S. (Cham: Springer). 10.1007/978-3-319-66682-2_14

[B36] Idris E. E. Bochow H. Ross H. Borriss R. (2004). Use of *Bacillus subtilis* as biocontrol agent. Phytohormone-like action of culture filtrates prepared from plant growth-promoting *Bacillus amyloliquefaciens* FZB24, FZB42, FZB45 and *Bacillus subtilis* FZB37. *J. Plant Dis. Prot.* 111 583–597. 10.2307/43215615

[B37] Ingvardsen C. Veierskov B. Joshi P. (2001). Immunohistochemical localisation of ubiquitin and the proteasome in sunflower (*Helianthus annuus* cv. Giganteus). *Planta* 213 333–341. 10.1007/s004250000511 11506355

[B38] Jammer A. Gasperl A. Luschin-Ebengreuth N. Heyneke E. Chu H. Cantero-Navarro E. (2015). Simple and robust determination of the activity signature of key carbohydrate metabolism enzymes for physiological phenotyping in model and crop plants. *J. Exp. Bot.* 66 5531–5542. 10.1093/jxb/erv228 26002973

[B39] Jebara S. Jebara M. Limam F. Aouani M. E. (2005). Changes in ascorbate peroxidase, catalase, guaiacol peroxidase and superoxide dismutase activities in common bean (*Phaseolus vulgaris*) nodules under salt stress. *J. Plant Physiol.* 162 929–936. 10.1016/j.jplph.2004.10.005 16146319

[B40] Kang S.-M. Khan A. L. Waqas M. Asaf S. Lee K.-E. Park Y.-G. (2019). Integrated phytohormone production by the plant growth-promoting rhizobacterium *Bacillus tequilensis* SSB07 induced thermotolerance in soybean. *J. Plant Interact.* 14 416–423. 10.1080/17429145.2019.1640294

[B41] Kour D. Rana K. L. Yadav N. Yadav A. N. Kumar A. Meena V. S. (2019). “Rhizospheric microbiomes: biodiversity, mechanisms of plant growth promotion, and biotechnological applications for sustainable agriculture,” in *Plant Growth Promoting Rhizobacteria for Agricultural Sustainability*, eds Kumar A. Meena V. (Singapore: Springer), 19–65. 10.1007/978-981-13-7553-8_2

[B42] Kumari B. Mallick M. A. Solanki M. K. Solanki A. C. Hora A. Guo W. (2019). “Plant growth promoting rhizobacteria (PGPR): modern prospects for sustainable agriculture,” in *Plant Health Under Biotic Stress*, eds Sayyed R. Z. Reddy M. S. Antonius S. (Singapore: Springer), 109–127. 10.1007/978-981-13-6040-4_6

[B43] LaFever R. E. Vogel B. S. Croteau R. (1994). Diterpenoid resin acid biosynthesis in Conifers: enzymatic cyclization of geranylgeranyl pyrophosphate to abietadiene, the precursor of abietic acid. *Arch. Biochem. Biophys.* 3131 139–149. 10.1006/abbi.1994.1370 8053674

[B44] Lata R. Gond S. K. (2019). “Plant growth-promoting microbes for abiotic stress tolerance in plants,” in *Role of Plant Growth Promoting Microorganisms in Sustainable Agriculture and Nanotechnology*, eds Choudhary K. K. Kumar A. Singh A. K. (Amsterdam: Elsevier), 89–105. 10.1016/b978-0-12-817004-5.00006-3

[B45] Liu T. (1971). *A Monograph of the Genus Abies.* Taipei: National Taiwan University, 608.

[B46] Liu X. Zhang H. Zhao Y. Feng Z. Li Q. Yang H.-Q. (2013). Auxin–ABA interaction controls seed dormancy. *Proc. Natl. Acad. Sci. U.S.A.* 110 15485–15490. 10.1073/pnas.1304651110 23986496 PMC3780901

[B47] Lübeck P. S. Alekhina I. A. Lübeck M. Bulat S. A. (1998). UP-PCR genotyping and rDNA analysis of *Ascochyta pisi* lib. *J. Phytopathol.* 146 51–55. 10.1111/j.1439-0434.1998.tb04749.x

[B48] Lucy M. Reed E. Glick B. R. (2004). Applications of free living plant growth-promoting rhizobacteria. *Antonie Van Leeuwenhoek* 86 1–25. 10.1023/B:ANTO.0000024903.10757.6e 15103234

[B49] Magel E. Einig W. Hampp R. (2000). Carbohydrates in trees. *Dev. Crop Sci.* 26 317–336. 10.1016/S0378-519X(00)80016-1

[B50] Marcos F. C. C. Iório R. P. F. Silveira A. P. D. Ribeiro R. V. Machado E. C. Lagôa A. M. M. A. (2016). Endophytic bacteria affect sugarcane physiology without changing plant growth. *Bragantia* 75 1–9. 10.1590/1678-4499.256

[B51] Martens H. J. Sørensen S. Burow M. Veierskov B. (2019). Characterization of top leader elongation in Nordmann fir (*Abies nordmanniana*). *J. Plant Growth Regul.* 38 1354–1361. 10.1007/s00344-019-09938-5

[B52] Martínez-Viveros O. Jorquera M. A. Crowley D. E. Gajardo G. Mora M. L. (2010). Mechanisms and practical considerations involved in plant growth promotion by Rhizobacteria. *J. Soil Sci. Plant Nutr.* 10 293–319. 10.4067/S0718-95162010000100006 27315006

[B53] Mercado-Blanco J. Abrantes I. Caracciolo A. B. Bevivino A. Ciancio A. Grenni P. (2018). Belowground microbiota and the health of tree crops. *Front. Microbiol.* 9:1006. 10.3389/fmicb.2018.01006 29922245 PMC5996133

[B54] Mhadhbi H. Jebara M. Limam F. Aouani M. E. (2004). Rhizobial strain involvement in plant growth, nodule protein composition and antioxidant enzyme activities of chickpea-rhizobia symbioses: modulation by salt stress. *Plant Physiol. Biochem.* 42 717–722. 10.1016/j.plaphy.2004.07.005 15474377

[B55] Nelson E. B. (2018). The seed microbiome: origins, interactions, and impacts. *Plant Soil* 422 7–34. 10.1007/s11104-017-3289-7

[B56] Nielsen U. B. Hansen J. K. Kromann H. K. (2011). Impact of site and provenance on economic return in Nordmann fir Christmas tree production. *Scand. J. For. Res.* 26 74–89. 10.1080/02827581.2010.526955

[B57] Olanrewaju O. S. Glick B. R. Babalola O. O. (2017). Mechanisms of action of plant growth promoting bacteria. *World J. Microbiol. Biotechnol.* 33:197. 10.1007/s11274-017-2364-9 28986676 PMC5686270

[B58] Ortíz-Castro R. Contreras-Cornejo H. A. Macías-Rodríguez L. López-Bucio J. (2009). The role of microbial signals in plant growth and development. *Plant Signal. Behav.* 4 701–712. 10.4161/psb.4.8.9047 19820333 PMC2801380

[B59] Overmann J. Abt B. Sikorski J. (2017). Present and future of culturing bacteria. *Annu. Rev. Microbiol.* 71 711–730. 10.1146/annurev-micro-090816-093449 28731846

[B60] Paulin M. M. Nicolaisen M. H. Jacobsen C. S. Gimsing A. L. Sørensen J. Bælum J. (2013). Improving Griffith’s protocol for co-extraction of microbial DNA and RNA in adsorptive soils. *Soil Biol. Biochem.* 63 37–49. 10.1016/j.soilbio.2013.02.007

[B61] Pindi P. K. Sultana T. Vootla P. K. (2014). Plant growth regulation of Bt-cotton through *Bacillus* species. *3 Biotech* 4 305–315. 10.1007/s13205-013-0154-0 28324434 PMC4026452

[B62] Polle A. Otter T. Seifert F. (1994). Apoplastic peroxidases and lignification in needles of Norway spruce (*Picea abies* L.). *Plant Physiol.* 106 53–60. 10.1104/pp.106.1.53 12232302 PMC159498

[B63] Qin S. J. Zhou W. J. Li Z. X. Lyu D. G. (2016). Effects of rhizobacteria on the respiration and growth of *Cerasus sachalinensis* Kom. seedlings. *Span. J. Agric. Res.* 14:e0803 10.5424/sjar/2016142-6848

[B64] Rais A. Jabeen Z. Shair F. Hafeez F. Y. Hassan M. N. (2017). *Bacillus* spp., a bio-control agent enhances the activity of antioxidant defense enzymes in rice against *Pyricularia oryzae*. *PLoS One* 12:e0187412. 10.1371/journal.pone.0187412 29161274 PMC5697883

[B65] Rasmussen H. N. Veierskov B. Hansen-Møller J. Nørbæk R. Nielsen B. N. (2009). Cytokinin profiles in the conifer tree *Abies nordmanniana*: whole-plant relations in year-round perspective. *J. Plant Growth Regul.* 28 154–166. 10.1007/s00344-009-9084-9

[B66] Rodríguez C. E. Mitter B. Barret M. Angela S. Compant S. (2018). Commentary: seed bacterial inhabitants and their routes of colonization. *Plant Soil* 422 129–134. 10.1007/s11104-017-3368-9

[B67] Rostamikia Y. Kouchaksaraei M. T. Asgharzadeh A. (2016). Effect of plant growth promoting rhizobacteria (PGPR) and cold stratification on seed germination and early growth of *Corylus avellana* L. *Austrian J. For. Sci.* 4 337–352. 10.1007/978-90-481-8661-7_63

[B68] Sakr S. Wang M. Dédaldéchamp F. Perez-Garcia M. D. Ogé L. Hamama L. (2018). The sugar-signaling hub: overview of regulators and interaction with the hormonal and metabolic network. *Int. J. Mol. Sci.* 19:2506. 10.3390/ijms19092506 30149541 PMC6165531

[B69] Sandhya V. Ali S. Z. Grover M. Reddy G. Venkateswarlu B. (2010a). Effect of plant growth promoting *Pseudomonas* spp. on compatible solutes, antioxidant status and plant growth of maize under drought stress. *Plant Growth Regul.* 62 21–30. 10.1007/s10725-010-9479-4

[B70] Sandhya V. Ali S. Z. Venkateswarlu B. Reddy G. Grover M. (2010b). Effect of osmotic stress on plant growth promoting *Pseudomonas* spp. *Arch. Microbiol.* 192 867–876. 10.1007/s00203-010-0613-5 20700582

[B71] São José J. F. B. Volpiano C. G. Vargas L. K. Hernandes M. A. S. Lisboa B. B. Schlindwein G. (2019). Influence of hot water on breaking dormancy, incubation temperature and rhizobial inoculation on germination of *Acacia mearnsii* seeds. *Aust. For.* 82 157–161. 10.1080/00049158.2019.1636350

[B72] Sasse J. Martinoia E. Northen T. (2018). Feed your friends: do plant exudates shape the root microbiome? *Trends Plant Sci.* 23 25–41. 10.1016/j.tplants.2017.09.003 29050989

[B73] Schiestl-Aalto P. Ryhti K. Mäkelä A. Peltoniemi M. Bäck J. Kulmala L. (2019). Analysis of the NSC storage dynamics in tree organs reveals the allocation to belowground symbionts in the framework of whole tree carbon balance. *Front. For. Glob. Change* 2:17 10.3389/ffgc.2019.00017

[B74] Seifert J. R. (2015). Growing Christmas trees. *Scott. For.* 55 231–233.

[B75] Shelake R. M. Pramanik D. Kim J.-Y. (2019). Exploration of plant-microbe interactions for sustainable agriculture in CRISPR Era. *Microorganisms* 7:269. 10.3390/microorganisms7080269 31426522 PMC6723455

[B76] Shishido M. Massicotte H. B. Chanway C. P. (1996). Effect of plant growth promoting *Bacillus* strains on pine and spruce seedling growth and mycorrhizal infection. *Ann. Bot.* 77 433–442. 10.1006/anbo.1996.0053

[B77] Sørensen M. T. Danielsen V. (2006). Effects of the plant growth regulator, chlormequat, on mammalian fertility. *Int. J. Androl.* 29 129–133. 10.1111/j.1365-2605.2005.00629.x 16466532

[B78] Vardharajula S. Ali S. Z. Grover M. Reddy G. Bandi V. (2011). Drought-tolerant plant growth promoting *Bacillus* spp.: effect on growth, osmolytes, and antioxidant status of maize under drought stress. *J. Plant Interact.* 6 1–14. 10.1080/17429145.2010.535178

[B79] Veierskov B. Ferguson I. B. (1991). Ubiquitin conjugating activity in leaves and isolated chloroplasts from *Avena sativa* L. during senescence. *J. Plant Physiol.* 138 608–613.

[B80] Vessey J. K. (2003). Plant growth promoting rhizobacteria as biofertilizers. *Plant Soil* 255 571–586. 10.1023/A:1026037216893

[B81] Wagner K. Krause K. Gallegos-Monterrosa R. Sammer D. Kovács Á. T. Kothe E. (2019). The ectomycorrhizospheric habitat of Norway spruce and *Tricholoma vaccinum*: promotion of plant growth and fitness by a rich microorganismic community. *Front. Microbiol.* 10:307. 10.3389/fmicb.2019.00307 30842767 PMC6391851

[B82] Weber R. Gessler A. Hoch G. (2019). High carbon storage in carbon-limited trees. *New Phytol.* 222 171–182. 10.1111/nph.15599 30451299

[B83] Wu C. H. Bernard S. M. Andersen G. L. Chen W. (2009). Developing microbe-plant interactions for applications in plant-growth promotion and disease control, production of useful compounds, remediation and carbon sequestration. *Microb. Biotechnol.* 2 428–440. 10.1111/j.1751-7915.2009.00109.x 21255275 PMC3815904

[B84] Xie X. Zhang H. Pare P. (2009). Sustained growth promotion in Arabidopsis with long-term exposure to the beneficial soil bacterium *Bacillus subtilis* (GB03). *Plant Signal. Behav.* 4 948–953. 10.4161/psb.4.10.9709 19826235 PMC2801358

[B85] Yang H. Puri A. Padda K. P. Chanway C. P. (2017). Substrate utilization by endophytic bacteria *Paenibacillus polymyxa* P2b-2R that may facilitate bacterial entrance and survival inside diverse plant hosts. *FACETS* 2 120–130.

[B86] Yang Y. Wang N. Guo X. Zhang Y. Ye B. (2017). Comparative analysis of bacterial community structure in the rhizosphere of maize by high-throughput pyrosequencing. *PLoS One* 12:e0178425. 10.1371/journal.pone.0178425 28542542 PMC5444823

[B87] Yoshimura K. Yabuta Y. Ishikawa T. Shigeoka S. (2000). Expression of spinach ascorbate peroxidase isoenzymes in response to oxidative stresses. *Plant Physiol.* 123 223–234. 10.1104/pp.123.1.223 10806239 PMC58996

[B88] Zulueta-Rodríguez R. Hernández-Montiel L. G. Murillo-Amador B. Rueda-Puente E. O. Capistrán L. L. Troyo-Diéguez E. (2015). Effect of hydropriming and biopriming on seed germination and growth of two Mexican fir tree species in danger of extinction. *Forests* 6 3109–3122. 10.3390/f6093109

