# Supplementary material for: Identification of Root-Associated Bacteria That Influence Plant Physiology, Increase Seed Germination, or Promote Growth of the Christmas Tree Species Abies nordmanniana
Source: Front Microbiol. 2020 Nov 17;11:566613. doi: 10.3389/fmicb.2020.566613 (PMC7705201; doi:10.3389/fmicb.2020.566613)
Supplement: Supplementary file 1 [file Data_Sheet_1.PDF]

SUPPLEMENTARY MATERIAL

Appendix 1: Carbohydrate determination in *A. nordmanniana* samples

Soluble carbohydrates were extracted by mixing approximately 125 mg (two replicates per sample) ground material with 3 ml 80% ethanol, 20 µl ribitol solution (25 mg/ml; internal standard) and incubation at 80°C for 10 min. The extraction was repeated twice, and the individual supernatants were combined. Extracts were supplemented with 3 ml dichloromethane and carefully mixed. After addition of 2 ml Milli-Q water, vortexing and incubation at 4°C overnight, extracts were centrifuged at 3000 × *g* for 10 min and the aqueous/alcohol phase was transferred to a new tube. After adding 2 ml Milli-Q water to the dichloromethane phase, the mixture was centrifuged, and the aqueous/alcohol phase was combined to the previously collected one. Subsequently, extracts were completely dried with compressed air and reconstituted in 5 ml Milli-Q water. The extracts were subjected to IC analysis to determine sucrose (Suc), glucose (Glc) and fructose (Fru). If needed, extracts were diluted depending on the concentration of the analytes.

For starch determination, the remaining plant material pellet was washed three times with Milli-Q water prior to drying. The pellet was resuspended in 3 ml 5 mM NaH_2PO_4 (pH 6.0) and 0.5 ml α-amylase solution (Termamyl 300L, Sigma-Aldrich) by vortexing. Samples were incubated for 60 min at 98°C and occasional vortexing. After cooling, samples were centrifuged for 10 min at 4000 × *g*. On ice, 100 µl of the supernatant were mixed with 850 µl NaAc buffer (50 mM NaAc, 15 mM $MgCl_2$, pH 4.6) and 50 µl amyloglucosidase solution (0.5 U/ml). The samples were vortexed, incubated for 60 min at 55°C and cooled afterwards. The extracts were subjected to IC analysis to determine Glc released from starch degradation. As controls for the starch determination (external standardization), 4 aliquots (50 mg) of potato flour were subjected to the identical protocol.To determine the carbohydrate amounts, extracts were analyzed on the integrated IC system 881 Compact IC pro (Metrohm Inula, Vienna). The extracts were loaded on an autosampler and 20 µl of each extract were injected twice on a Metrosep Carb 2 - Guard/4.0 and a Metrosep Carb 2 - 150/4.0 column combination at 37°C with 100 mM NaOH (supplemented with 16.6 mM NaAc) as eluent at a flow rate of 0.5 ml/min, followed by detection with an 896 Professional Detector – Amperometry (Metrohm Inula) in pulsed amperiometric detection mode.

Apart from the individual amounts of carbohydrates and starch determined, following parameters were derived from these data: The hexose: sucrose ratio was determined, where hexose levels are represented by the sum of Glc and Fru. The sum of Glc, Fru and Suc served as an estimate of total

soluble carbohydrates (∑ sol. CH). The sum of Glc, Fru, Suc and starch served as an estimate of nonstructural carbohydrate amounts (∑ NSC).

Supplementary Table 1. Identified root-associated bacterial strains isolated from *Abies nordmanniana*. Plants from one, two and three years of growth, were collected from Danish and German field and greenhouse nurseries. Sequences obtained after partial 16S rRNA gene sequencing, were matched in the GenBank nucleotide database using the Basic Local Alignment Search Tool (BLAST). In the "Isolated from plant sample name" column; gh means greenhouse samples, F means field samples, and 1, 2, 3 means plant age of one, two and three years old, respectively.

**Blast match (97-99% id.)**	**Strain ID**	**Isolated from plant sample name**
Acetobacter sp.	*Acetobacter* sp._s2	1_gh
Agrobacterium rubi	*Agrobacterium* sp._s5	1_gh
Bacillus acidiceler	*Bacillus* sp._s7	1_gh
Bacillus arbutinivorans	*Bacillus* sp._s9	1_gh
Bacillus arybhattai	*Bacillus* sp._s10	1_gh
Bacillus arybhattai	Bacillus sp._s31	3_F
*Bacillus cereus**	*Bacillus* sp._s11*	1_gh
*Bacillus cereus**	*Bacillus* sp._s14*	1_gh
*Bacillus cereus**	*Bacillus* sp._s15*	2_gh
*Bacillus cereus**	*Bacillus* sp._s18*	3_F
Bacillus circulans	*Bacillus* sp._s17	1_gh
Bacillus licheniformis	*Bacillus* sp._s20	2_gh
Bacillus licheniformis	*Bacillus* sp._s52	3_F
Bacillus marisflavi	*Bacillus* sp._s22	2_gh
Bacillus megaterium	*Bacillus* sp._s19	2_gh
Bacillus megaterium	*Bacillus* sp._s23	3_F
Bacillus megaterium	*Bacillus* sp._s24	2_F
Bacillus megaterium	*Bacillus* sp._s25	3_F
Bacillus megaterium	*Bacillus* sp._s26	3_F
Bacillus megaterium	*Bacillus* sp._s27	3_F
Bacillus megaterium	*Bacillus* sp._s28	3_F
Bacillus mycoides	*Bacillus* sp._s29	2_gh
Bacillus mycoides	*Bacillus* sp._s32	3_F
Bacillus mycoides	*Bacillus* sp._s39	3_F
Bacillus mycoides	*Bacillus* sp._s43	3_F
Bacillus paralicheniformis	*Bacillus* sp._s34	1_F
Bacillus paralicheniformis	*Bacillus* sp._s35	3_F
Bacillus pumillus	*Bacillus* sp._s6	3_F
Bacillus pumilus	*Bacillus* sp._s36	3_F
Bacillus safensis	*Bacillus* sp._s1	1_F
Bacillus simplex	*Bacillus* sp._s30	3_F
Bacillus simplex	*Bacillus* sp._s38	2_F
Bacillus simplex	*Bacillus* sp._s41	2_F
Bacillus simplex	*Bacillus* sp._s42	3_F

Bacillus simplex	*Bacillus* sp._s44	2_gh
Bacillus simplex	*Bacillus* sp._s47	2_gh
Bacillus simplex	*Bacillus* sp._s48	3_F
Bacillus simplex	*Bacillus* sp._s49	3_F
Bacillus sp.	*Bacillus* sp._s13	2_F
Bacillus sp.	*Bacillus* sp._s16	2_F
Bacillus sp.	*Bacillus* sp._s33	2_gh
Bacillus sp.	*Bacillus* sp._s45	3_F
Bacillus sp.	*Bacillus* sp._s50	3_F
Bacillus sp.	*Bacillus* sp._s51	1_F
Bacillus sp.	*Bacillus* sp._s53	1_F
Bacillus sp.	*Bacillus* sp._s54	2_F
Bacillus sp.	*Bacillus* sp._s55	2_F
Bacillus sp.	*Bacillus* sp._s56	3_F
Bacillus sp.	*Bacillus* sp._s57	3_F
Bacillus sp.	*Bacillus* sp._s58	1_gh
Bacillus sp.	*Bacillus* sp._s59	1_gh
Bacillus sp.	*Bacillus* sp._s60	2_gh
Bacillus sp.	*Bacillus* sp._s61	2_gh
Bacillus sp.	*Bacillus* sp._s62	2_gh
Bacillus sp.	*Bacillus* sp._s63	2_gh
Bacillus sp.	*Bacillus* sp._s64	2_gh
Bacillus sp.	*Bacillus* sp._s65	3_F
Bacillus sp.	*Bacillus* sp._s66	3_F
Bacillus sp.	*Bacillus* sp._s67	3_F
Bacillus sp.	*Bacillus* sp._s68	3_F
Bacillus sp.	*Bacillus* sp._s69	3_F
Bacillus sp.	*Bacillus* sp._s70	3_F
Bacillus sp.	*Bacillus* sp._s71	3_F
Bacillus sp.	*Bacillus* sp._s72	3_F
Bacillus sp.	*Bacillus* sp._s73	3_F
Bacillus sp.	*Bacillus* sp._s8	1_F
Bacillus subtilis	*Bacillus* sp._s74	3_F
Bacillus subtillis	*Bacillus* sp._s75	3_F
Bacillus thioparans	*Bacillus* sp._s76	3_F
Bacillus thioparans	*Bacillus* sp._s77	1_gh
Bacillus thuringiensis	*Bacillus* sp._s78	3_F
Bacillus thuringiensis	*Bacillus* sp._s79	3_F
Bacillus velezensis	*Bacillus* sp._s80	1_gh
Curtobacterium flaccumfaciens	*Curtobacterium* sp._s46	3_F
Erwinia sp.	*Erwinia* sp._s81	1_F
Erwinia sp.	*Erwinia* sp._s82	2_gh
Erwinia sp.	*Erwinia* sp._s83	3_F
Erwinia sp.	*Erwinia* sp._s84	3_F
Exiguobacterium sp.	*Exiguobacterium* sp._s85	2_F
Lysinibacillus sp.	*Lysinibacillus* sp._s86	3_F
Lysinibacillus sp.	*Lysinibacillus* sp._s87	3_F
Microbacterium hydrocarbonoxidans	*Microbacterium* sp._s88	2_F
Microbacterium sp.	*Microbacterium* sp._s89	2_F
Micrococcus yunnanensis	*Micrococcus* sp._s90	1_gh
Ochrobacterium sp.	*Ochrobacterium* sp._s91	2_gh
Paenibacillus glycanilyticus	*Paenibacillus* sp._s92	1_gh
Paenibacillus lautus	*Paenibacillus* sp._s93	2_gh
Paenibacillus lautus	*Paenibacillus* sp._s94	3_F
Paenibacillus polymyxa	*Paenibacillus* sp._s95	2_F
Paenibacillus polymyxa	*Paenibacillus* sp._s96	2_F

Paenibacillus sp.	*Paenibacillus* sp._s100	3_F
Paenibacillus sp.	*Paenibacillus* sp._s12	2_gh
Paenibacillus sp.	*Paenibacillus* sp._s97	3_F
Paenibacillus sp.	*Paenibacillus* sp._s98	2_gh
Paenibacillus sp.	*Paenibacillus* sp._s99	2_gh
Paenibacillus tundrae	*Paenibacillus* sp._s101	3_F
Paenibacillus xtyanexedens	*Paenibacillus* sp._s102	3_F
Paenicibacillus sp.	*Paenicibacillus* sp._s103	3_F
Paenicibacillus sp.	*Paenicibacillus* sp._s37	3_F
Paenicibacillus sp.	*Paenicibacillus* sp._s40	3_F
Pantoea rodasii	*Pantoea* sp._s104	3_F
Pseudomonas fluorescens	*Pseudomonas* sp._s105	3_F
Pseudomonas fluorescens	*Pseudomonas* sp._s106	3_F
Pseudomonas putida	*Pseudomonas* sp._s107	3_F
Pseudomonas putida	*Pseudomonas* sp._s108	3_F
Pseudomonas reinekei	*Pseudomonas* sp._s109	3_F
Pseudomonas sp.	*Pseudomonas* sp._s110	3_F
Pseudomonas sp.	*Pseudomonas* sp._s111	2_gh
Pseudomonas sp.	*Pseudomonas* sp._s112	3_F
Pseudomonas sp.	*Pseudomonas* sp._s113	3_F
Pseudomonas sp.	*Pseudomonas* sp._s114	3_F
Pseudomonas sp.	*Pseudomonas* sp._s115	3_F
Pseudomonas sp.	*Pseudomonas* sp._s116	3_F
Pseudomonas sp.	*Pseudomonas* sp._s21	1_F
Pseudomonas sp.	Pseudomonas sp._s3	3_F
Pseudomonas vranovensis	*Pseudomonas* sp._s117	2_F
Psychrobacillus Psychrodurans	*Psychrobacillus* sp._s118	2_F
Rahnella aquatilis	*Rahnella* sp._s119	3_F
Rhodococcus sp.	*Rhodococcus* sp._s120	3_F
Sporosarcina luteola	*Sporosarcina* sp._s121	3_F
Stenophomonas rhizophila	*Stenophomonas* sp._s122	1_gh
Sterophomonas sp.	*Sterophomonas* sp._s123	1_F
*Straphylococcus pateuri**	*Straphylococcus* sp._s124*	3_F
Streptomycets lavendulae	*Streptomycets* sp._s4	3_F
Streptomycets sp.	*Streptomycets* sp._s125	1_gh
Viridibacillus arvi	*Viridibacillus* arvi_s126	1_gh

Highlighted in blue are the bacterial strains that were used for the in-vitro seed germination test, and seed germination percentages from experiments with the strains in red are shown in the results section. *Strains not used for experiments due to risk for human health.

Supplementary Table 2. Mineral composition in the field soil where the treated seedlings were to be planted. 100 g samples were taken 5 different places in the field and pooled before being analyzed. Eurofins Agro Testing Denmark A/S (www.eurofins.dk) performed the mineral analysis.

mg (100mg DW soil)$^{-1}$											
K	P	Ca	Mg	S	Cu	Mn	Zn	B	Na	Cl	pH
7.7	3.9	0.3	5.0	7.9	4.6	6.7	2.2	3.5	0.7	0.3	6.0

Supplementary Table 3. Levels of the auxin indole-acetic acid (IAA) produced by pure culture of bacterial strains grown on liquid media M9 (minimal nutrient) supplemented with 200 µl of L-tryptophan (+) and in absence of L- tryptophan (-), controls without bacterial inoculum were performed. Values represents the mean of three technical replicates.

**Treatments**	**L- tryptophan in media**	**IAA (pmol g FW^{-1})**
Control (-)	(-)	0
	(+)	0
Bacillus sp._s7	(-)	0
	(+)	1
Bacillus sp._s45	(-)	0
	(+)	0
Bacillus sp._s50	(-)	8
	(+)	125
Paenibacillus sp._s12	(-)	17
	(+)	66
Paenibacillus sp._s37	(-)	5
	(+)	244
Paenibacillus sp._s40	(-)	5
	(+)	112

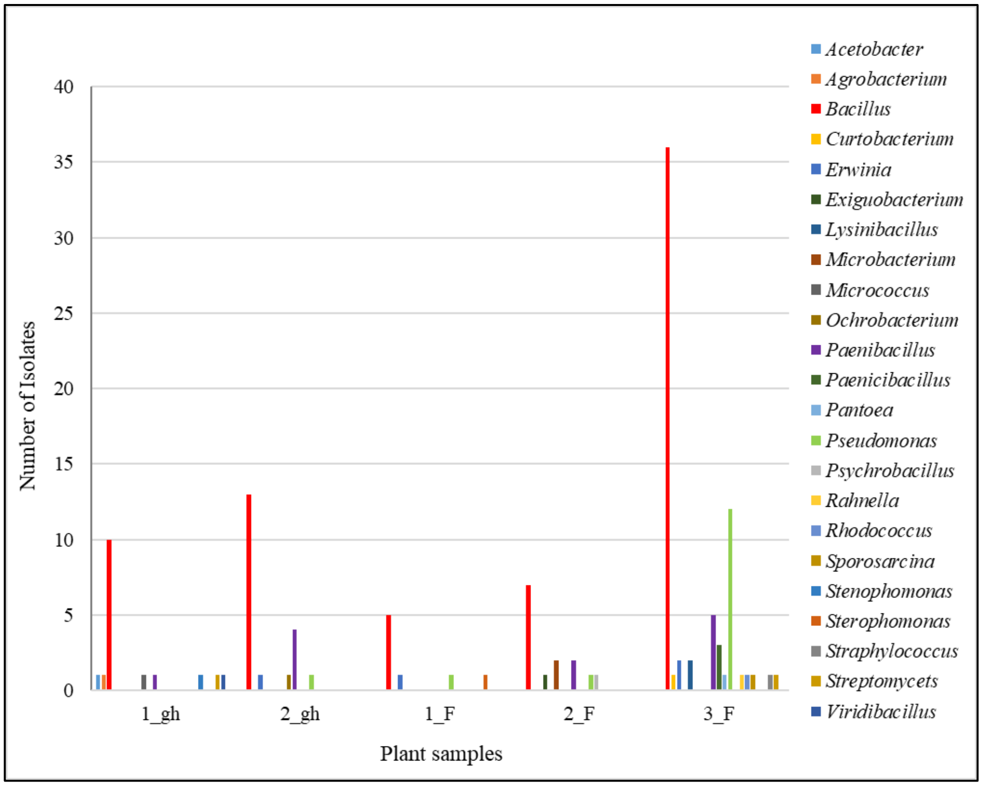

Supplementary Figure S1. Number of isolates per genus level from *A. nordmanniana* plants of one and two years collected at the greenhouse nursery (1_gh; 2_gh), and plants of one, two and three years collected at the field nursery (1_F; 2_F; 3_F).

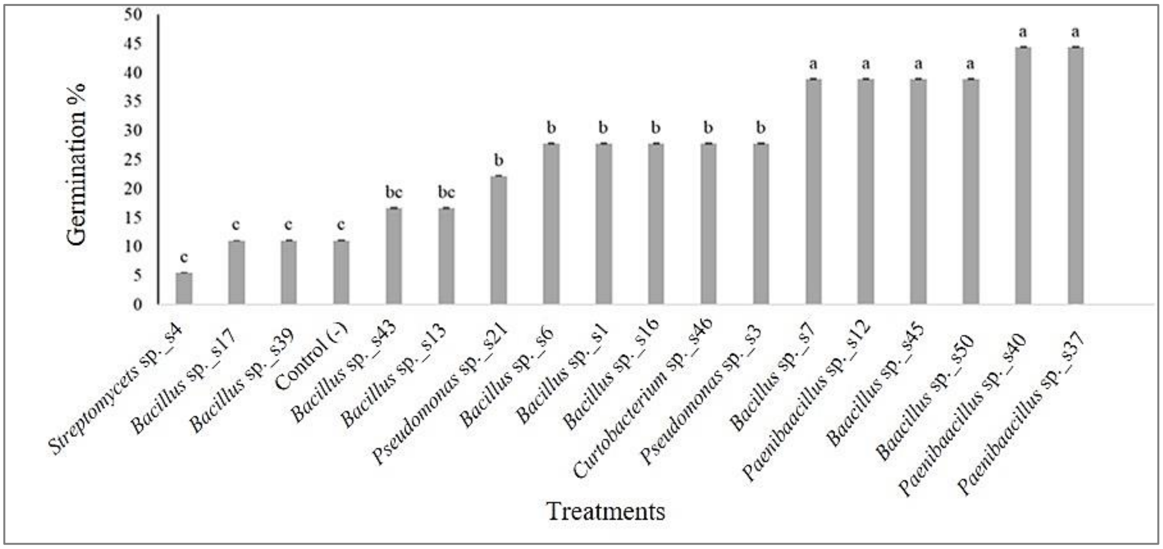

Supplementary Figure S2. Germination percentage of seeds of *A. nordmanniana* inoculated with different root-associated bacterial strains isolated from *A. nordmanniana* plants collected in nurseries in Denmark and in Germany. The figure shows the average percentage of germination of three replicates per treatment and three independent repetitions. Only the treatments (17 bacterial strains + negative control) with at least two germinating seeds are shown. Different letters indicate significantly different means at $P<0.05$ (Post-hoc test). Each bar represents mean ± SE.

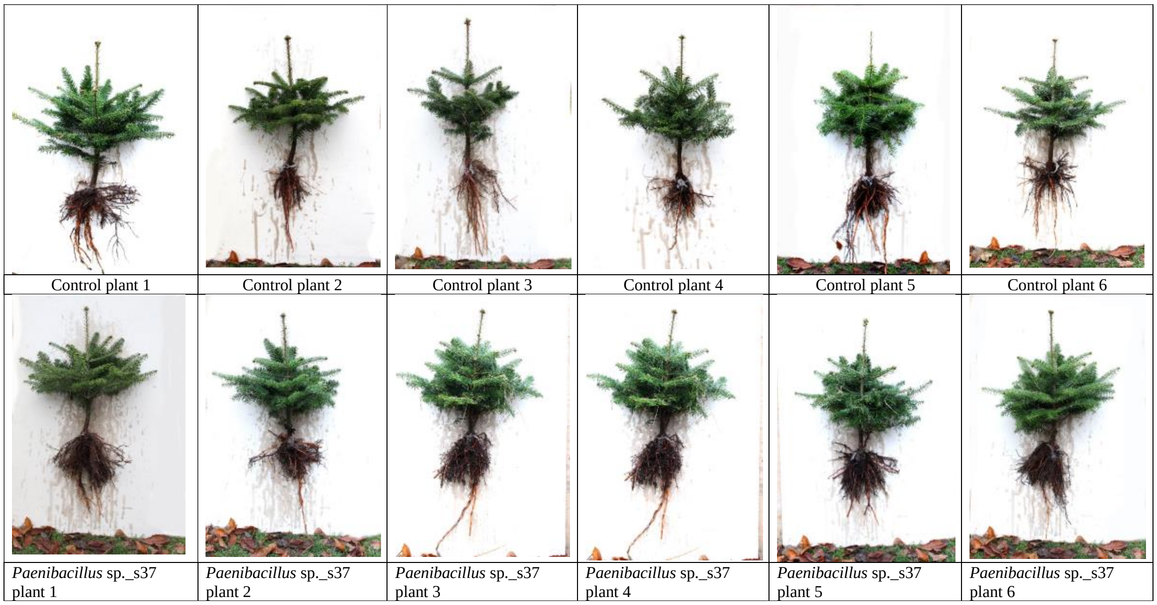

Supplementary Figure S3. Root development of two-years old *A. nordmanniana* plants grown under field conditions. The top panels show six control plants that had not been inoculated, while the bottom panels show six plants that had been seed inoculated with *Paenibacillus* sp. strain s37.
